# Structural and functional analysis of lignostilbene dioxygenases from *Sphingobium* sp. SYK-6

**DOI:** 10.1016/j.jbc.2021.100758

**Published:** 2021-05-07

**Authors:** Eugene Kuatsjah, Anson C.K. Chan, Rui Katahira, Stefan J. Haugen, Gregg T. Beckham, Michael E.P. Murphy, Lindsay D. Eltis

**Affiliations:** 1 Department of Microbiology and Immunology, Life Sciences Institute, The University of British Columbia, Vancouver, Canada; 2 Renewable Resources and Enabling Sciences Center, National Renewable Energy Laboratory, Golden, Colorado, USA; 3 Center for Bioenergy Innovation, Oak Ridge National Laboratory, Oak Ridge Tennessee, USA; 4 BioProducts Institute, The University of British Columbia, Vancouver, Canada

**Keywords:** aromatic catabolism, lignostilbene, carotenoid cleavage oxygenase, lignin degradation, bacterial catabolism, 5-formylferulate, 4-[(*E*)-2-carboxyethenyl]-2-formyl-6-methoxyphenolate, CCD, carotenoid cleavage dioxygenases, DCA, dehydrodiconiferyl alcohol, DCA-S, 3-(4-hydroxy-3-(4-hydroxy-3-methoxystyryl)-5-methoxyphenyl) acrylate, DCM, dichloromethane, DMF, dimethyformamide, GC-MS, gas chromatography–mass spectrometry, HEPPS, 4-(2-hydroxyethyl)-1-piperazinepropanesulfonic acid, *I*, ionic strength, ICP-MS, inductively coupled plasma–mass spectrometry, LSD, lignostilbene-α,β-dioxygenase, PDB, protein data bank, RMSD, root-mean-square deviation, SEC-MALS, size-exclusion chromatography–multiangle light scattering, SYK-6, *Sphingobium* sp. SYK-6, TMY1009, *Sphingomonas paucimobilis* TMY1009, *t*_R_, retention time

## Abstract

Lignostilbene-α,β-dioxygenases (LSDs) are iron-dependent oxygenases involved in the catabolism of lignin-derived stilbenes. *Sphingobium* sp. SYK-6 contains eight LSD homologs with undetermined physiological roles. To investigate which homologs are involved in the catabolism of dehydrodiconiferyl alcohol (DCA), derived from β-5 linked lignin subunits, we heterologously produced the enzymes and screened their activities in lysates. The seven soluble enzymes all cleaved lignostilbene, but only LSD2, LSD3, and LSD4 exhibited high specific activity for 3-(4-hydroxy-3-(4-hydroxy-3-methoxystyryl)-5-methoxyphenyl) acrylate (DCA-S) relative to lignostilbene. LSD4 catalyzed the cleavage of DCA-S to 5-formylferulate and vanillin and cleaved lignostilbene and DCA-S (∼10^6^ M^−1^ s^−1^) with tenfold greater specificity than pterostilbene and resveratrol. X-ray crystal structures of native LSD4 and the catalytically inactive cobalt-substituted Co-LSD4 at 1.45 Å resolution revealed the same fold, metal ion coordination, and edge-to-edge dimeric structure as observed in related enzymes. Key catalytic residues, Phe-59, Tyr-101, and Lys-134, were also conserved. Structures of Co-LSD4·vanillin, Co-LSD4·lignostilbene, and Co-LSD4·DCA-S complexes revealed that Ser-283 forms a hydrogen bond with the hydroxyl group of the ferulyl portion of DCA-S. This residue is conserved in LSD2 and LSD4 but is alanine in LSD3. Substitution of Ser-283 with Ala minimally affected the specificity of LSD4 for either lignostilbene or DCA-S. By contrast, substitution with phenylalanine, as occurs in LSD5 and LSD6, reduced the specificity of the enzyme for both substrates by an order of magnitude. This study expands our understanding of an LSD critical to DCA catabolism as well as the physiological roles of other LSDs and their determinants of substrate specificity.

The bacterial catabolism of aromatic compounds has attracted considerable attention recently due to its biocatalytic potential in valorizing lignin (1, 2, 3, 4, 5). Lignin is a heterogeneous aromatic polymer found in plant cell walls and comprises up to 30% of lignocellulosic biomass (2, 6). Studies have established that the sustainability of next-generation biorefineries depends on the valorization of all components of lignocellulose, including lignin (2, 6, 7). Due in part to their ability to catabolize a wide variety of aromatic compounds and their genetic tractability, bacteria provide an entry to developing biocatalysts to upgrade lignin depolymerization products to compounds such as muconic acid, β-ketoadipic acid, and 2-pyrone-4,6-dicarboxylic acid, which can be used to produce polymers (2, 8). Developing bacteria as lignin-valorizing biocatalysts requires elucidating and optimizing enzymes and pathways that catabolize lignin-derived aromatic compounds.

*Sphingobium* sp. SYK-6 (SYK-6 hereafter) is among the best characterized bacteria able to catabolize lignin-derived aromatic compounds (2, 4). Initially isolated from the waste water of a kraft pulp mill, SYK-6 has been shown to grow on compounds containing many of the intersubunit linkages found in lignin, including β-aryl ethers (β-*O*-4 linkage), pinoresinol (β-β), 2,2′-dihydroxy-3,3′-dimethoxy-5,5′-dicarboxybiphenyl (5-5), 1,2-bis(4-hydroxy-3-methoxyphenyl)-1,3-propanediol (β-1), and dehydrodiconiferyl alcohol (β-5, DCA), catabolizing them *via* vanillate and syringate (9, 10). Indeed, the bacterium appears to be wired for growth on aromatic compounds, growing better on vanillin than glucose, and deriving significant amounts of NAD(P)H from vanillin catabolism (11). In addition, SYK-6 has been used to biocatalytically convert hardwood lignin hydrolyzates to muconate, which can be hydrogenated to adipate, and SYK-6 enzymes have been used to engineer a biocatalyst to convert lignosulfonate to 2-pyrone-4,6-dicarboxylic acid (12, 13). Nevertheless, many of the enzymes responsible for the catabolism of lignin-derived aromatic compounds by SYK-6 have yet to be characterized, particularly for those containing β-1 and β-5 linkages.

In the catabolism of DCA by SYK-6, DCA is oxidized to a stilbene, 3-(4-hydroxy-3-(4-hydroxy-3-methoxystyryl)-5-methoxyphenyl) acrylate (DCA-S), which is then proposed to be cleaved by a lignostilbene-α,β-dioxygenase (LSD) (Fig. 1) (14). More particularly, the two nonphenolic alcohols of DCA are successively oxidized to aldehydes, then carboxylates (14, 15). The terminal allylic alcohol of DCA is oxidized to an acrylate *via* an aldehyde intermediate, 3-(2-(4-hydroxy-3-methoxyphenyl)-3-(hydroxymethyl)-7-methoxy-2,3-dihydrobenzofuran-5-yl)acrylaldehyde (DCA-L), to ultimately yield 3-(2-(4-hydroxy-3-methoxyphenyl)-3-(hydroxymethyl)-7-methoxy-2,3-dihydrobenzofuran-5-yl)acrylate (DCA-C) (14, 15). The presumed dehydrogenases responsible for these steps have yet to be identified. By contrast, the enzymes that oxidize the methyl alcohol attached to the coumaran moiety of DCA-C have been partially characterized. PhcC, PhcD, and an unidentified dehydrogenase catalyze the oxidation of DCA-C to the two stereoisomers of 5-(2-carboxyvinyl)-2-(4-hydroxy-3-methoxy-phenyl)-7-methoxy-2,3-dihydrobenzofuran-3-carboxylate (DCA-CC) *via* an aldehyde intermediate, 3-(3-formyl-2-(4-hydroxy-3-methoxyphenyl)-7-methoxy-2,3-dihydrobenzofuran-5-yl)acrylate (DCA-CL) (16). PhcC and PhcD are glucose-methanol-choline oxidoreductases, and their reactions are coupled to respiration (15). The two stereoisomers of DCA-CC are decarboxylated to form DCA-S in a reaction involving PhcF, PhcG, and, to some extent, PhcH (16).

**Figure 1:**
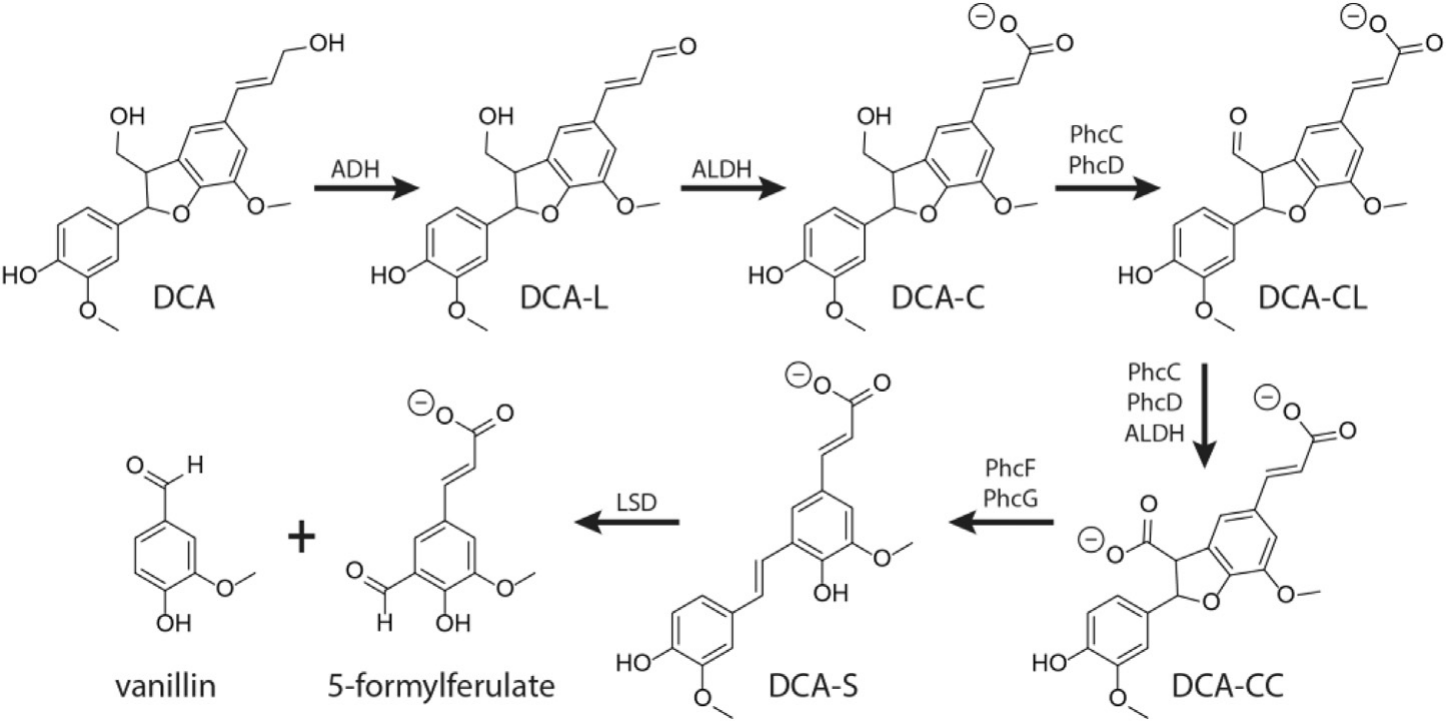
**Proposed catabolism of DCA in SYK-6.** ADH and ALDH represent unidentified alcohol and aldehyde dehydrogenases, respectively. Adapted from Takahashi *et al.* (16).

LSDs are nonheme iron enzymes that catalyze the O_2_-dependent cleavage of *trans*-4-hydroxystilbenes to yield two aromatic aldehydes (17, 18). In the case of DCA-S, LSD-catalyzed cleavage is expected to yield vanillate and 5-formylferulate (18, 19). LSDs belong to the family of carotenoid cleavage dioxygenases (CCD), best characterized in eukaryotes whose structural fold is characterized by a seven-bladed β-propeller fold (20, 21). The active site Fe^2+^ resides at the core of the β-propeller and is ligated by four histidines, three of which are hydrogen-bonded to acidic residues (21, 22). These seven residues are remarkably conserved in all CCDs characterized to date (21, 22). The coordination sphere of the active site Fe^2+^ forms a distorted bipyramidal configuration consisting of the four histidines and a solvent species. The sixth site is unoccupied, partly due to occlusion by a nearby hydrophobic residue (23). Due to their relative ease of preparation and the relative solubility of their substrates, LSDs have served as important models for understanding CCDs and their catalytic mechanism (20, 24).

In bacteria, LSDs have been assigned catabolic and protective roles. For example, the catabolism of β-1-diaryl propane by *Sphingomonas paucimobilis* TMY1009 (TMY1009 hereafter), which, like SYK-6, grows on a variety of lignin-derived aromatic compounds, involves a stilbenoid intermediate in the form of lignostilbene (25, 26). TMY1009 harbors four isoforms of LSD, which cleave lignostilbene to two equivalents of vanillin (19, 27). Similarly, a rhizosphere-associated bacterium, *Acinetobacter* sp., grows on resveratrol as a sole carbon source (28, 29). LSDs have also been proposed to function as virulence factors in plant pathogens, potentially conferring protection against stilbenoid-based phytoalexins (30). Interestingly, SYK-6 harbors eight LSD homologs (4). Of these, LSD1 and LSD2 share 99% amino acid sequence identity with LsdA_TMY1009_ and LsdB_TMY1009_ of TMY1009, respectively, while the other six share 35 to 58% amino acid sequence identity. It is unclear why SYK-6 harbors so many LSD homologs.

To elucidate the physiological roles of the eight LSD homologs of SYK-6, we produced the enzymes in *Escherichia coli* and investigated their relative abilities to cleave lignostilbene and DCA-S. Based on this screen, we further characterized the structure and function of LSD4. Chromatographic and mass spectrometry analyses were employed to identify LSD-catalyzed reaction products of DCA-S. Steady-state kinetic studies were performed to evaluate the substrate specificity of LSD4 against a variety of stilbenoids. X-ray crystal structures were determined, including that of LSD4 bound to each of two substrates and a product. Oligonucleotide-directed mutagenesis was employed to evaluate the role of Ser-283 in determining the substrate specificity of the enzyme. The results are discussed with respect to the physiological roles of LSDs and the catabolism of lignin-derived aromatic compounds by SYK-6.

## Results

### Phylogeny of SYK-6 LSDs

**Figure 2:**
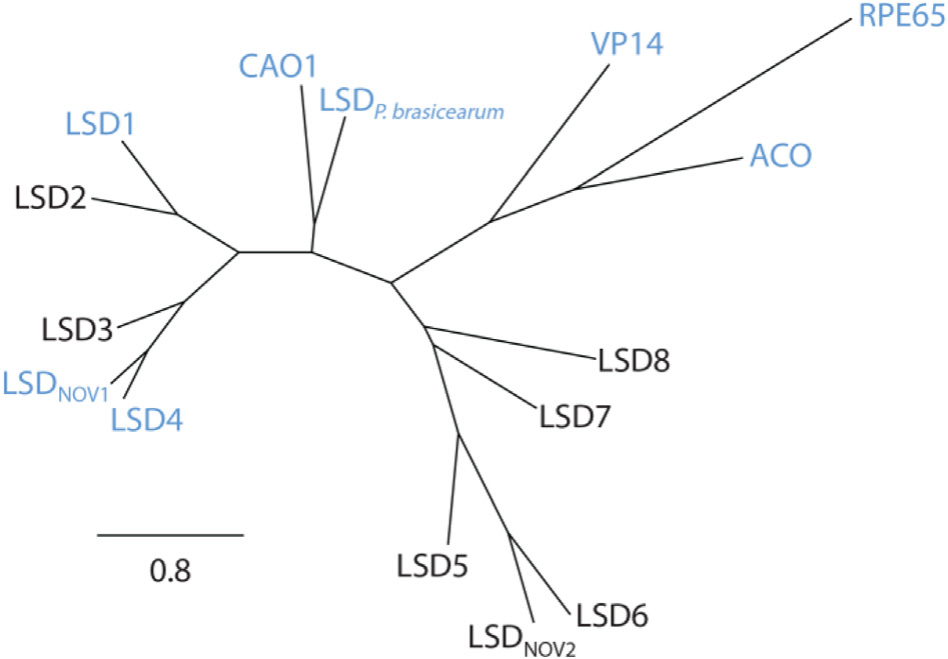
**Radial phylogram of CCDs and LSDs.** Maximum likelihood tree was calculated used a structure-based sequence alignment. LSDs from *Sphingobium sp*. SYK-6: LSD1 (SLG_37540), LSD2 (SLG_36640), LSD3 (SLG_11300), LSD4 (SLG_12860; PDB entry: 6XMA), LSD5 (SLG_27970), LSD6 (SLG_12580), LSD7 (SLG_09440), and LSD8 (SLG_27300). Other enzymes are: LSD_NOV1_ (Saro_0802, PDB entry: 5J53) and LSD_NOV2_ (Saro_2809) from *Novosphingobium aromaticivorans* DSM12444; CAO1 (XP_961764, PDB entry: 5U8X) from *Neurospora crassa* OR74A; LSD (WP_025212951, PDB entry: 5V2D) from *Pseudomonas brassicearum*; apocarotenoid-15,15’-oxygenase ACO (P74334, PDB entry: 2BIW) from *Synechocystis* sp. PCC 6803 substrain Kazusa; 9-*cis*-epoxycarotenoid dioxygenase VP14 (O24592, PDB entry: 3NPE) from *Zea mays*; and retinol isomerase RPE65 (Q28175, PDB entry: 3FSN) from *Bos taurus*. LsdA_TMY1009_ (Q53353, PDB entry: 6OJW) and LsdB_TMY1009_ (Q52008) from *Sphingomonas paucimobilis* TMY1009 were omitted from the figure due to their ≥98% shared amino acid sequence identity with to LSD1 and LSD2, respectively. Twenty-four N-terminal residues of LsdC from *S. paucimobilis* TMY1009 are identical to those of LSD4. Structurally characterized proteins are in blue. LSD1–LSD8 of SYK-6 correspond to LsdH, LsdG, LsdC, LsdD, LsdF, LsdA, LsdB, and LsdE, respectively, in Kamimura *et al.* (34).

To better understand the relationship of the eight LSD homologs of SYK-6 (4), we performed phylogenetic analyses of select LSDs and CCDs using a structure-guided alignment (Fig. 2). Importantly, all eight homologs of SYK-6 are most closely related to characterized stilbenoid-cleaving dioxygenases than to other CCDs. Moreover, LSD1 through 4 comprise a clade of enzymes that includes LsdA_TMY1009_ and LsdB_TMY1009_ and LSD_NOV1_ of *Novosphingobium aromaticivorans* DSM12444. The enzymes of this clade share at least 57% amino acid sequence identity.

### The lignostilbene-cleaving activities of SYK-6 enzymes

**Figure 3:**
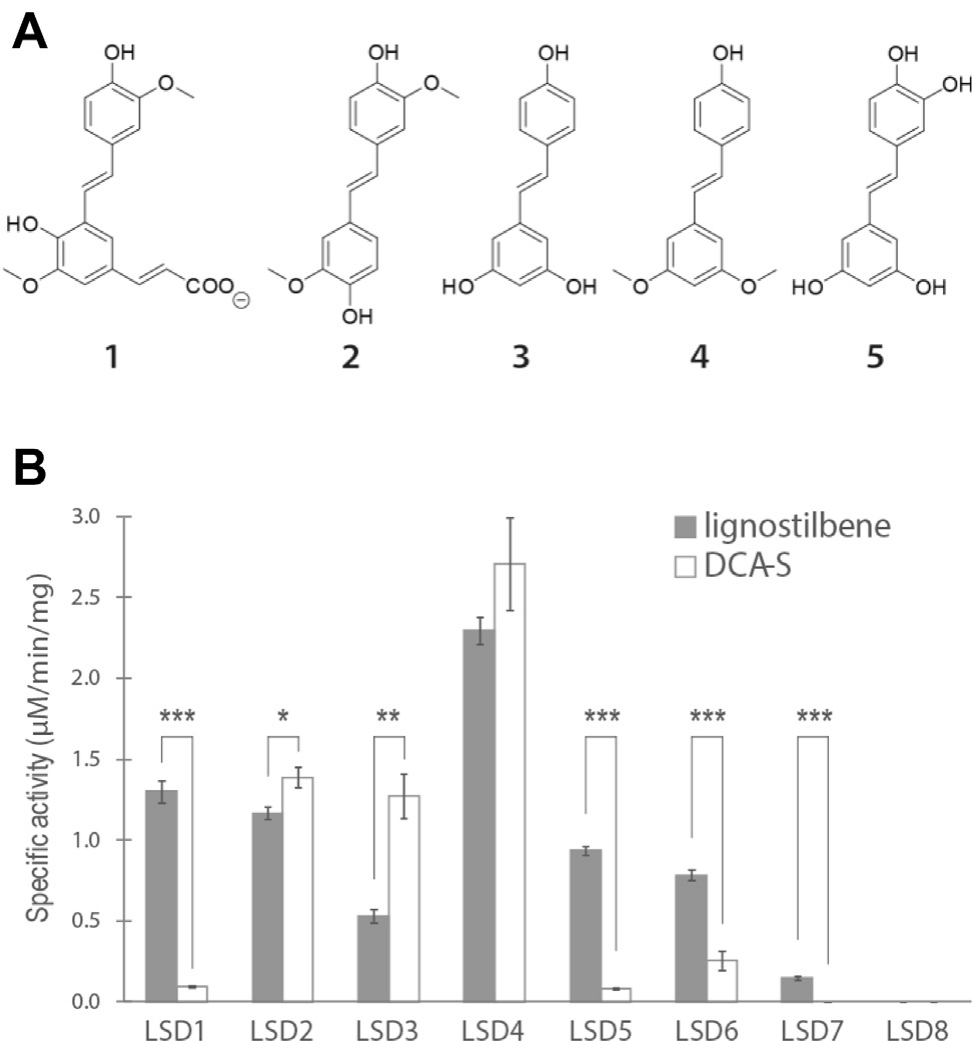
**Compounds used in this study and the specific activities of different LSDs.***A*, DCA-S (1), lignostilbene (2), resveratrol (3), pterostilbene (4), and piceatannol (5). *B*, relative activity of the lysates against lignostilbene (*gray*) and DCA-S (*white*) as measured in an oxygraph assay using 100 μM of each substrate. The error bars represent standard deviations of three technical replicates. ∗, ∗∗, and ∗∗∗ denote statistical significance with *p*-value ≤0.05, 0.005, and 0.0005, respectively, based on the two-tailed *t*-test.

To evaluate which of the eight LSD homologs of SYK-6 are involved in the catabolism of DCA, we screened their relative abilities to cleave DCA-S and lignostilbene (Fig. 3*A*). To this end, the genes encoding each homolog were cloned and heterologously expressed in *E. coli*. SDS-PAGE analysis revealed that seven of the homologs (LSD1–LSD7) were produced as soluble proteins when cells were grown on LB medium supplemented with iron, although LSD1 and LSD2 required the coproduction of chaperones GroEL and GroES (Fig. S1). By contrast, LSD8 was insoluble even in the presence of the chaperones. In an oxygraph assay performed using 100 μM stilbenoid substrate, cell lysates containing LSD1, LSD5, LSD6, or LSD7 preferentially cleaved lignostilbene over DCA-S (Fig. 3*B*). By contrast, lysates containing LSD2, LSD3, or LSD4 preferentially cleaved DCA-S over lignostilbene. Finally, lysates prepared from cells expressing LSD8 did not detectably cleave either stilbene, consistent with the apparent insolubility of this enzyme. Overall, lysate containing LSD4 displayed approximately twofold more DCA-S-cleavage activity than any other lysate. However, lysate containing LSD3 displayed the highest DCA-S-cleavage activity relative to lignostilbene cleavage.

### Preparation of LSD4 and Co-LSD4

To further characterize LSD4, the heterologously produced protein was purified to >99% apparent homogeneity using hydrophobic interaction and anion exchange chromatographies. Approximately 20 mg of purified LSD4 was obtained per liter of cell culture and contained ≥0.9 equivalent of iron per protomer as measured using a Ferene S-based assay. Size-exclusion chromatography–multiangle light scattering (SEC-MALS) analysis indicated that LSD4 exists in solution as a mono-dispersed species with a mass of 106.3 ± 0.4 kDa, consistent with a dimer (Fig. S2; LSD4 protomer: 54 kDa).

To facilitate the structural characterization of LSD4·substrate complexes, we generated Co-LSD4 in which the catalytic Fe^2+^ was substituted with Co^2+^. This metal ion substitution in CAO1 yielded an inactive but isostructural enzyme (31, 32). Co-LSD4 was produced by growing cells producing LSD4 in a minimal medium supplemented with Ca^2+^, Mg^2+^, and Co^2+^ as the only divalent metal ions (31, 32). Purification yielded ∼8 mg of protein per liter of cell culture. Inductively coupled plasma–mass spectrometry (ICP-MS) analysis revealed that each protomer of Co-LSD4 contained 0.92 ± 0.06 equivalents of Co^2+^ and ∼0.02 equivalents of Fe^2+^. Co-LSD4 had a faint purple hue and a λ_max_ of ∼480 nm (Fig. S3).

### DCA-S cleavage products

**Figure 4:**
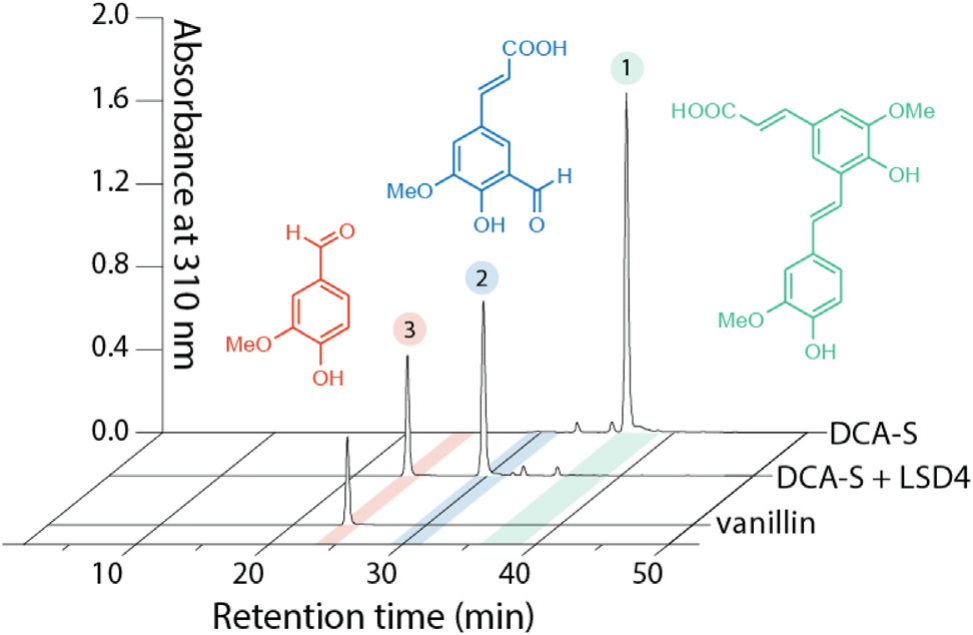
**LSD4-catalyzed DCA-S cleavage products.** HPLC traces, labeled on the *right*, are of DCA-S, DCA-S incubated with LSD4, and vanillin. Numbered peaks corresponding to DCA-S (1), 5-formylferulate (2), and vanillin (3) are highlighted with *green*, *blue*, and *red*, respectively.

To identify products of the LSD4-catalyzed cleavage of DCA-S, the substrate and enzyme were incubated for ∼15 min at room temperature in potassium phosphate (ionic strength (*I*) = 0.1 M), pH 7.5. HPLC analysis of the reaction revealed the depletion of DCA-S (retention time (*t*_R_), ∼38 min) and the appearance of two products (*t*_R_ ∼25 min and ∼30 min; Fig. 4). The first peak eluted with the same *t*_R_ as vanillin and, when analyzed using gas chromatography–mass spectrometry (GC-MS), had an *m*/*z* value of 224.1 (Fig. S4), consistent with trimethylsilylyated vanillin. The second HPLC peak had an *m*/*z* value of 366.2 when analyzed using GC-MS, consistent with doubly trimethylsilylyated 5-formylferulate. HPLC-based assays further revealed that the LSD4-catalyzed cleavage of DCA-S and lignostilbene yielded 1.1 ± 0.3 mol and 2.6 ± 0.2 mol vanillin per mol O_2_ consumed, respectively. Overall, these analyses demonstrated that LSD4 catalyzes the cleavage of DCA-S to vanillin and 5-formylferulate, consistent with the proposed DCA catabolic pathway of SYK-6 (14, 16), and that the cleavage of lignostilbene and DCA-S is well coupled to O_2_ consumption.

### Substrate specificity

To investigate the substrate specificity of LSD4, we used an oxygraph assay to evaluate the enzyme’s apparent specificity (*k*_cat_/*K*_M_) for a variety of stilbenes (HEPES (*I* = 0.1 M), pH 7.5 at 25 °C). For all tested stilbenes, the dependence of the initial reaction rate on substrate concentration obeyed Michaelis–Menten kinetics. As summarized in Table 1, LSD4 exhibited slightly higher specificity for lignostilbene over DCA-S and cleaved both with approximately tenfold higher apparent specificity than either pterostilbene or resveratrol. Interestingly, LSD4 was relatively unreactive toward piceatannol, cleaving it with a specific activity <1% that of lignostilbene. Subsequent analysis of the steady-state utilization of O_2_ revealed *K*_M_ values comparable with those of LsdA_TMY1009_ (33). Accordingly, the *k*_cat_ values for lignostilbene or DCA-S under O_2_-saturating conditions were estimated to be twice those measured using air-saturated buffer (Table 2). However, the *k*_cat_/*K*_M O2_ values were similar in the presence of lignostilbene and DCA-S.

**Table 1:** Apparent steady-state kinetic parameters of LSD4 for different substrates^a^

Substrate	$k_{\text{cat}}^{app}$	$K_{\text{M}}^{app}$	$k_{\text{cat}}^{app}/K_{\text{M}}^{app}$
	s^−1^	μM	× 10^5^ s^−1^ M^−1^
lignostilbene	18 ± 1	13 ± 1	14 ± 1
DCA-S	11 ± 1	12 ± 1	9 ± 1
pterostilbene	1.9 ± 0.1	15 ± 2	1.3 ± 0.1
resveratrol	2.1 ± 0.1	22 ± 2	0.9 ± 0.1

a Experiments were performed using HEPES (*I* = 0.1 M), pH 7.5, at 25 °C. Parameters were calculated using a minimum of 20 data points at various substrate concentrations and were obtained using air-saturated buffer and are thus apparent.

**Table 2:** Apparent steady-state kinetic parameters of LSD4 for different substrates^a^

Substrate	Enzyme	$k_{\text{cat}}^{app}$	$K_{\text{M}}^{app}$	$k_{\text{cat}}^{app}/K_{\text{M}}^{app}$	$k_{\text{cat}}$^b^	$K_{\text{M}\_\text{O}2}$	$k_{\text{cat}}/K_{\text{M}\_\text{O}2}$
		s^−1^	μM	× 10^5^ s^−1^ M^−1^	s^−1^	μM	× 10^5^ s^−1^ M^−1^
lignostilbene	WT	18 ± 1	13 ± 1	14 ± 1	39 ± 2	320 ± 20	1.2 ± 0.1
	S283A	15.3 ± 0.4	10 ± 1	15 ± 1	20 ± 1	400 ± 30	0.50 ± 0.04
	S283F	0.14 ± 0.01	8.5 ± 0.7	0.16 ± 0.02	n.d.^c^	n.d.	n.d.
DCA-S	WT	11 ± 1	12 ± 1	9 ± 1	26 ± 1	220 ± 20	1.2 ± 0.1
	S283A	4.1 ± 0.1	4.6 ± 0.5	9 ± 1	7.8 ± 0.6	410 ± 60	0.19 ± 0.03
	S283F	0.035 ± 0.001	2.7 ± 0.5	0.13 ± 0.05	n.d.	n.d.	n.d.

a Experiments were performed using HEPES (*I* = 0.1 M), pH 7.5, at 25 °C. Parameters were calculated using a minimum of 20 data points at various substrate concentrations. Apparent parameters for stilbene substrates were obtained using air-saturated buffer. Parameters obtained for O_2_, including *k*_cat_ values, were determined using 100 μM lignostilbene or DCA-S.

b Calculated from saturating amounts of O_2_ and stilbene.

c Not determined.

To investigate whether LSD4 is subject to product inhibition, we evaluated the ability of vanillin to inhibit the cleavage of lignostilbene. Vanillin functioned as a mixed-type inhibitor with *K*_ic_ and *K*_iu_ values of 30 ± 8 μM and 60 ± 7 μM (Fig. S5), respectively. As these values are approximately twofold higher than the apparent *K*_M_ values for lignostilbene and DCA-S, this suggests that product inhibition does not occur to a significant extent. In addition, if *K*_ic_ is taken as an approximation of *K*_d_, and *k*_on_ is diffusion-controlled, then the *K*_ic_ value further suggests that the product release is not rate-limiting.

Finally, incubating lignostilbene with Co-LSD4 led to rates of O_2_ consumption that were barely above background. This is consistent with Co-LSD4 being inactive, with residual activity in the preparation the result of trace Fe-LSD4 as noted above (Fig. S6).

### Structure of LSD4

**Figure 5:**
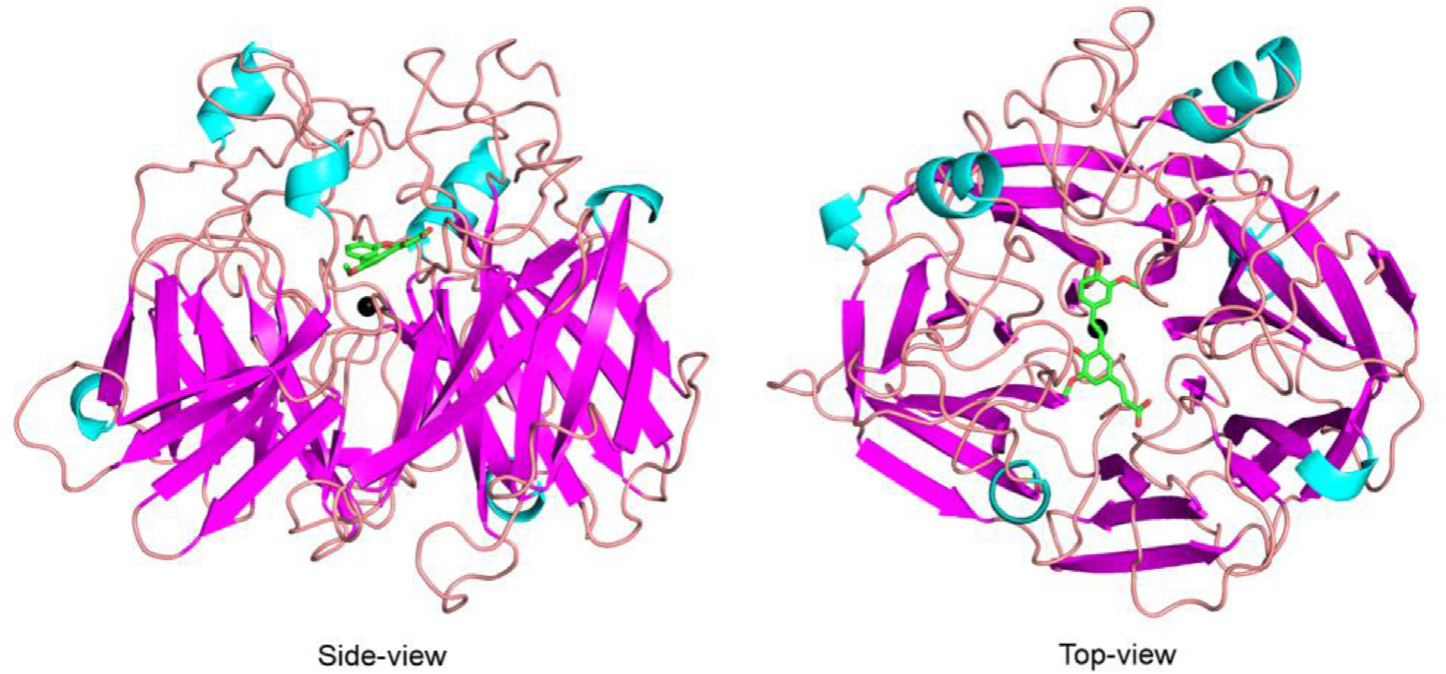
**Structure of the LSD4 protomer.** Side and top views of LSD4 protomer (PDB entry: 6XM7). The α-helices and β-strands are *light blue* and *magenta*, respectively. Carbon and oxygen atoms of the DCA-S are *green* and *red*, respectively. Fe^2+^ ion is shown as a *black sphere*.

**Figure 6:**
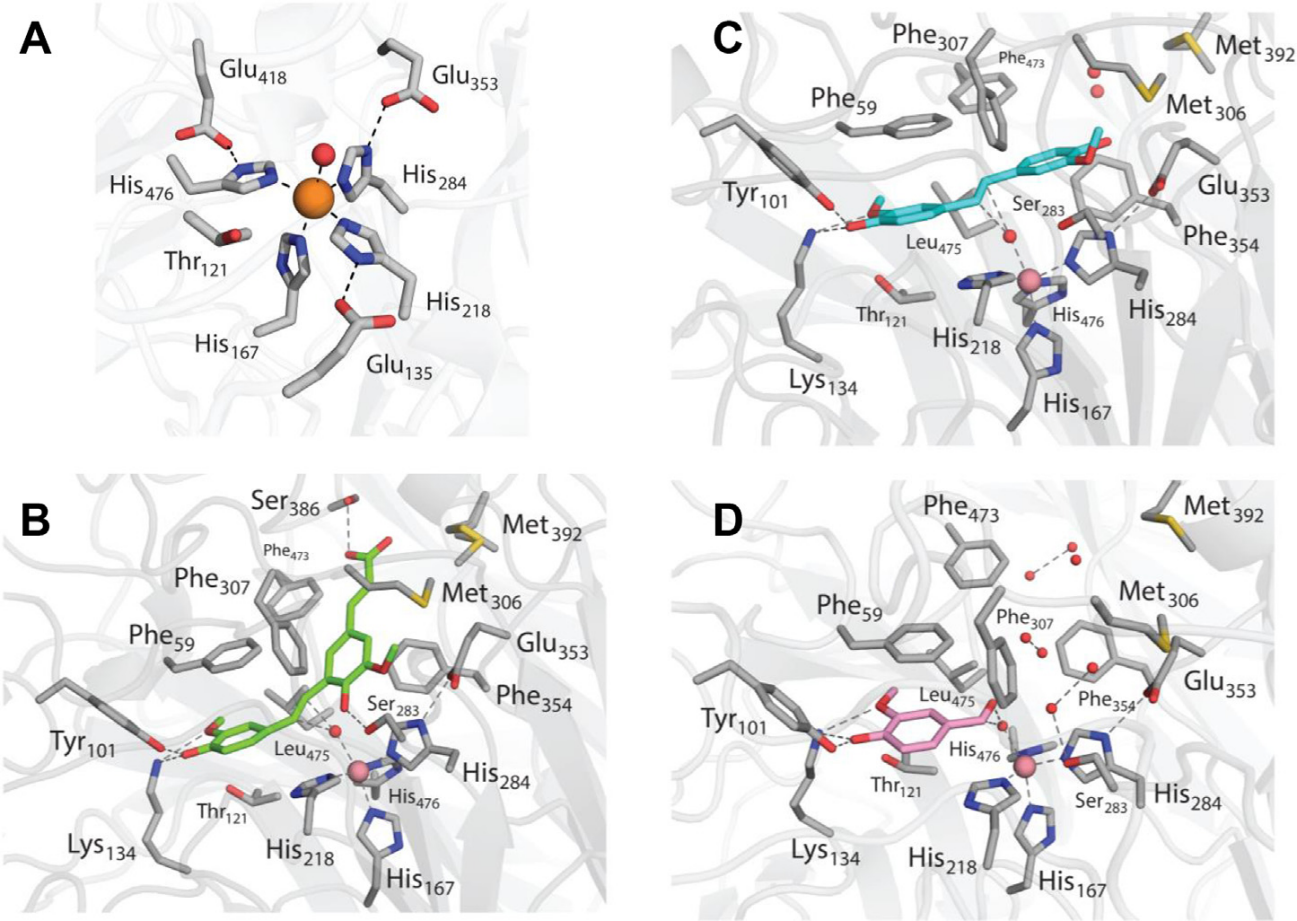
**The active site of LSD4 in different states of the enzyme.***A*, the Fe^2+^ coordination sphere in the resting state enzyme (PDB entry: 6XMA). LSD4 complexed with (*B*) DCA-S, (*C*) lignostilbene, and (*D*) vanillin (PDB entry: 6XM7, 6XM8, and 6XM9, respectively). LSD4 residues and organic ligands are represented as *sticks*. LSD4 residues, DCA-S, lignostilbene, and vanillin are colored *gray*, *green*, *cyan*, and *pink*, respectively. The Fe^2+^ ion, Co^2+^ ion and water molecules are represented as *orange*, *pink*, and *red spheres*, respectively. Metal–ligand bonds and hydrogen bonds (≤3.0 Å) are indicated using *dashed lines*.

To investigate the structural determinants of substrate specificity in LSD4, we determined the enzyme’s X-ray crystal structure. LSD4 was crystallized in space group *I*222 with a single protomer in the asymmetric unit and solved to 1.45 Å resolution (Table S1). The overall structure of LSD4 is typical of CCDs (21): a seven-bladed β-propeller fold with a cap-like feature on one face formed by extended loops (Fig. 5). Consistent with the phylogenetic analysis, LSD4 is most similar structurally to NOV1 (Protein Data Bank (PDB) entry 5J53), sharing a root-mean-square deviation (RMSD) of 0.4 Å over 479 aligned Cα atoms (24). As in all structurally characterized CCDs, the metal ion in LSD4 resides near the central axis of the β-propeller at the interface with the cap. The loops of the cap form a channel to the substrate-binding site adjacent to the metal. The bound Fe^2+^ has a distorted bipyramidal coordination sphere and is ligated by four conserved histidines (His-167, 218, 284, and 476) and a solvent molecule (Fig. 6*A*). The Fe^2+^-N bonds are of similar length, averaging 2.1 Å (Table S1). The sixth coordination position, across from His-284, is occluded by Thr-121 and is vacant. Three of the ligands, His-218, His-284, and His-476, are hydrogen-bonded to Glu-135, Glu-353, and Glu-418, respectively. The latter are conserved as acidic residues in CCDs.

The LSD4 dimer observed in solution can be reconstructed through crystallographic symmetry (Fig. S7). The interface of the LSD4 dimer is quite small, involving 32 residues with a surface area of ∼992 Å^2^, which constitutes ∼5% of the total solvent-accessible surface area of the protein. Indeed, an analysis using PDBePISA did not predict a stable quaternary structure in LSD4. The dimer interaction arranges the protomers in a manner similar to what has been reported in LsdA_TMY1009_ and CAO1, where an edge-to-edge interaction between the first propeller blade of each protomer creates a ten-stranded antiparallel β-sheet (32, 33). In LSD4, β1-strand of the first propeller blade is shorter than that in LsdA_TMY1009_ and CAO1, such that the interacting strands are offset with respect to each other in the dimer. Nevertheless, the interface in LSD4 is formed in large part by residues 16 to 28 that includes the β1-strand. Within this strand, Cys-21 is positioned such that a disulfide bond could be formed across the interface under oxic conditions. However, no such bond was observed in the structures, which were prepared aerobically and in the absence of reducing agents. Additional key interactions at the interface involve hydrogen bonds between Ala-2 and His-3 with Asn-25, Leu-26, and His-28 of the other protomer and between His-445 from both protomers. Among these residues, Ala-2 and His-3 are conserved in LsdA_TMY1009_ and LsdB_TMY1009_, while Leu-26 is a leucine and isoleucine, respectively.

### Structure of ligand-bound LSD4

To explore the nature of substrate binding in LSD4, we generated binary complexes with different stilbenoids. Attempts to cocrystallize the enzyme and substrate were unsuccessful. Further, attempts to soak crystals with substrates anaerobically yielded only low-resolution data sets. Therefore, we prepared catalytically inactive Co-LSD4, a strategy that yielded ligand-bound structures of CAO1 (31, 32). Co-LSD4 crystallized under similar conditions as native enzyme and the two structures were nearly identical, with an RMSD of 0.1 Å over 471 aligned Cα atoms. The coordination geometry of the active site metal ion was also highly similar in the two variants (Table S1). Anaerobic incubation of Co-LSD4 crystals with either lignostilbene or DCA-S yielded complexes of these ligands, while aerobic incubation with lignostilbene yielded a Co-LSD4·vanillin complex. The crystals were isomorphous with the native apo LSD4 crystals (Table S2). Further, the overall structure of LSD4 in the complexes was essentially identical to that of the resting state enzyme with an RMSD of ≤0.2 Å over at least 473 aligned Cα atoms. The presence of the stilbenoid or vanillin within the cap’s channel (Fig. 5) and adjacent to the catalytic metal ion was confirmed by omit difference density maps and modeled at full occupancy (Fig. S8). The high resolution of the structures permitted unambiguous determination of ligand orientation based on density fitting. In all three complexes, the coordination geometry of the active site metal ion was highly similar to that of LSD4 and Co-LSD4 (Table S1), and the coordinated solvent species was modeled at full occupancy.

In the Co-LSD4·DCA-S complex (Fig. 6*B*), the stilbenoid substrate is positioned in the active site such that the two carbon atoms of the substrate’s vinyl group are ∼4.3 Å from the cobalt ion. The solvent ligand is also present, between the substrate and the Co^2+^, ∼2.8 Å from both C atoms. There is sufficient space between the metal ion and the stilbenoid substrate for O_2_ to displace the solvent ligand as suggested for NOV1 and other CCDs (24, 32). The guaiacyl moiety of DCA-S interacts with conserved active site residues Phe-59, Tyr-101, and Lys-134 in the same manner as observed in related enzymes (32, 33). These interactions are primarily defined by the 4-hydroxyl group of the guaiacyl moiety, which forms hydrogen bonds with Tyr-101 and Lys-134, and by the phenolic ring of the guaiacyl group, which forms a π–π stacking interaction with Phe-59. These interactions are conserved in the Co-LSD4·lignostilbene and Co-LSD4·vanillin complexes. In all three structures, Lys-134 also interacts with the guaiacyl methoxy group and Asn-120. The latter is part of a hydrogen bonding network with Phe-59 and Gly-61 that occurs in the ligand-free structures. In contrast to the guaiacyl moiety, the density attributed to the ferulyl moiety of DCA-S is more diffuse, particularly for those portions of the molecule further away from the metal ion (Fig. S8*A*). For example, the average B-factors associated with the guaiacyl and the acrylate portions of DCA-S are 25 and 49 Å^2^, respectively. Similarly, the B-factors of the residues that interact with the acrylate group, Ser-386 and Ala-387, are higher than those of other active site residues. Indeed, the loop on which Ser-386 and Ala-387 are located appears to be flexible and was not fully modeled in all other structures due to poor density. Finally, the hydroxyl group of the ferulyl moiety of DCA-S forms a hydrogen bond with Ser-283. Sequence alignment with other LSD homologs revealed that this residue is conserved in LSD2, LSD4, and NOV1, but is glycine in CAO1, LSD_Pbra_, LSD1, and LSD7; alanine in LSD3; phenylalanine in LSD5 and LSD6; and isoleucine in LSD8. Another notable feature of the LSD4·DCA-S complex is that the double bond to be cleaved in the substrate is distorted ∼15° from planarity.

In the Co-LSD4·lignostilbene complex (Fig. 6*C*), the proximal guaiacyl group of lignostilbene interacts with the active site residues as described above for the Co-LSD4·DCA-S complex. As in the latter structure, the electron density of the distal aromatic moiety is more disperse (Fig. S8*B*), with the fitted atoms of the distal ring having an average B-factor twice that of the proximal ring (52 *versus* 26 Å^2^). In contrast to the proximal group, the distal guaiacyl of lignostilbene interacts more weakly with hydrophobic residues in the active site (Phe-354, Leu-475, Phe-307) and only participates in a single hydrogen bonding interaction between the C4’ hydroxyl and Glu-353 (3.2 Å). A possible second contributing factor to this observation might be partial cleavage of the substrate as observed previously (24, 32). Therefore, although the double bond of the bound lignostilbene is modeled planar, some twist is possible.

Incubation of Co-LSD4 with lignostilbene under aerobic conditions for ∼16 h at 8 °C yielded an E·vanillin complex (Fig. 6*D*). The slow cleavage of lignostilbene is presumably due to trace amounts of native LSD4 in preparations of Co-LSD4. The vanillin product equilibrated to active sites of Co-LSD4 and is modeled at full occupancy. The vanillin is bound in the active site in a manner very similar to that of the guaiacyl ring of the substrate in the Co-LSD4·DCA-S complex. The aldehyde group of vanillin interacts with two solvent molecules, one of which is the metal-bound species. Overall, the orientations of the substrates and product in the various LSD4 complexes are in agreement with structures of homologous complexes (24, 32, 33).

### Active-site variants

To evaluate the role of Ser-283 in determining the substrate specificity of LSD4, we substituted this residue with alanine and phenylalanine. Alanine occurs in this position in LSD3, which preferentially cleaves DCA-S over lignostilbene, while phenylalanine occurs in LSD5 and LSD6, which preferentially cleave lignostilbene over DCA-S. The two LSD4 variants were purified similarly as WT LSD4 and contained a full complement of iron. In both variants, substrate cleavage was well coupled to O_2_ consumption: for the S283A variant, the stoichiometry of vanillin produced per O_2_ consumed was 1.2 ± 0.1 and 2.3 ± 0.4 for DCA-S and lignostilbene, respectively. For the S283F variant, these variants were 1.2 ± 0.2 and 2.1 ± 0.2, respectively.

As summarized in Table 2, these substitutions did not alter the relative specificity of LSD4 for DCA-S relative to lignostilbene. Thus, the apparent *k*_cat_/*K*_M_ values of the S283A variant for the two substrates were comparable with those of the WT as both apparent turnover numbers and Michaelis constants decreased proportionally. This substitution also reduced the reactivity of the enzyme with O_2_, with *k*_cat_/*K*_M O2_ values being 20 to 40% those of wild-type LSD4. As this effect was similar in magnitude for the two substrates, it did not alter the conclusion that the substitution did not affect LSD4’s relative specificity for lignostilbene and DCA-S. Substitution of Ser-283 with phenylalanine dramatically lowered the *k*_cat_ values of LSD4 for the two substrates. Nevertheless, the relative magnitude of the *k*_cat_/*K*_M_ values was similar to those of WT LSD4.

## Discussion

In this study, we identified three LSDs from SYK-6 that catalyze the efficient cleavage of DCA-S with respect to lignostilbene: LSD2, LSD3, and LSD4. By contrast, four of the other five LSD homologs do not. These observations are consistent with the very recent study of LSDs in SYK-6 by Kamimura *et al.* (34). This substrate preference largely corresponds to the phylogeny of the LSDs inasmuch as the three DCA-S-cleaving enzymes cluster together (Fig. 2). Furthermore, these results agree with previous work on homologous enzymes from TMY1009 (19, 27). Notably, LSD-III and LSD-IV from TMY1009, which correspond to homodimers of LsdB and LsdC, respectively, both efficiently cleaved DCA-S (19, 27). As noted above, LsdB shares 99% amino acid sequence with LSD2. Further, LsdC is likely very similar to LSD4 as 24 N-terminal residues of the two proteins are identical (27). Nevertheless, the two enzymes diverge to some extent as LsdC had approximately twofold greater specificity for DCA-S than lignostilbene (27), in contrast to LSD4’s similar specificities for the two stilbenes. Unfortunately, the amino acid sequence of LsdC is not available, preventing further comparison at this time.

The substrate specificity, structural and mutagenesis studies are consistent with each other in that interactions between LSD4 and the proximal ring of the stilbenoid are more important determinants of the specificity of the enzyme than interactions with the distal ring. More particularly, the specificity data establish that LSD4 has a strong preference for substrates with a guaiacyl ring that can bind in the proximal position. The poor reactivity of LSD4 with piceatannol indicates that the guaiacyl ring cannot be substituted with a catechol. Importantly, piceatannol is a very good substrate for CAO1 and NOV2 (35). Further evidence for the importance of the guaiacyl ring is provided by LSDs from TMY1009 and *Pseudomonas putida* IE27, which cleave eugenol to vanillin (19, 36). Consistent with the specificity data, the structures of the Co-LSD4·DCA-S and Co-LSD4·lignostilbene complexes revealed a lack of specific polar interactions between the enzyme and the distal ring. It is thus unsurprising that Ser-283 is not a major determinant of specificity for DCA-S in LSD4 despite the interaction of this residue with the ferulyl moiety of DCA-S in the Co-LSD4·DCA-S complex. Indeed, glycine and alanine occur at this position in LSD2 and LSD3, two other DCA-S-cleaving enzymes. Presumably, phenylalanine at this position occludes binding site for the distal ring of the substrate. Nevertheless, the relative inability of LSD1 and LsdA_TMY1009_ to cleave DCA-S is somewhat surprising given that these enzymes cluster with enzymes that do. Comparing the structures of the LsdA_TMY1009_·phenylazophenol and LSD4·DCA-S complexes suggests that Phe-305 and Phe-307 of LsdA_TMY1009_/LSD1 may sterically clash with the acrylate moiety of DCA-S. The equivalent residues in LSD4, Phe-307, and Phe-309 are positioned differently due to differences in the backbones and adopted rotamers. It is unclear whether the positioning of these residues is the sole determinant of LSD1’s specificity for lignostilbene.

The current studies indicate that bacterial LSDs dimerize in the same “edge-to-edge” manner and that the specific interactions observed in LSD4 are likely to mediate dimerization in NOV1 and LSD-IV of TMY1009, which, as noted above, consists of a dimer of LsdC (27). A surface charge analysis of the LSD4 interface shows that it consists primarily of polar residues (Fig. S9) and its relatively small area (∼900 Å^2^) in LSD4 and NOV1 suggests that dimerization may be transient. Nevertheless, LSD4 exists almost exclusively as a dimer in solution. Furthermore, although CAO1, LsdA_TMY1009_, LsdB_TMY1009_, and LSD_Pbra_ also form dimers (19, 27, 32), the residues that mediate dimerization are poorly conserved. Indeed, the dimer interface in CAO1, LsdA_TMY1009_, and LSD_Pbra_ is larger (∼1400 Å^2^) than in NOV1 and LSD4 (Table S2). Notably, the CAO1 interface hosts the most extensive interactions with roughly double the H-bond interactions that were predicted for LsdA_TMY1009_ and LSD_Pbra_. Apart from CAO1, dimerization is mediated by approximately a dozen H-bonds regardless of the size of the interface and the identity of the H-bond donor and acceptors.

The function of dimerization in LSD is unclear. Dimerization is unlikely to impact the catalytic efficiency as the binding of the stilbenoid substrate has no significant impact on the overall structure of the enzyme or the dimerization interface. Moreover, the lack of conservation in dimer-mediated residues likely prevents the formation of heterodimers except in rare cases, such as LsdA_TMY1009_ and LsdB_TMY1009_, which have nearly identical N-terminal sequences (19). Among other CCDs, dimerization has only been reported in RPE65, responsible for the isomerization of all-*trans*-retinyl ester to 11-*cis*-retinol in the visual cycle of animals (37). In bovine RPE65, the dimerization is mediated by a stretch of ∼30 residues near the C-terminus of the protomer, which form two fairly hydrophobic contact points (37). However, the structural element responsible for this dimerization appears to be exclusive to vertebrate CCDs (37).

It remains unclear which LSD is responsible for DCA catabolism in SYK-6. In a very recent study, Kamimura *et al.* (34) established that genes encoding LSD2, LSD4, LSD6, and LSD7 were upregulated during growth on DCA, of which *lsdD*, encoding LSD4 (see Fig. 2 for nomenclature used by Kamimura *et al.*), was the most abundant transcript. Further, a Δ*lsdD* mutant of SYK-6 had no DCA-S cleavage activity when grown in the presence of vanillate, whereas mutants lacking either LSD2 or LSD6 had cleavage activities similar to wild-type SYK-6. Unfortunately, testing the growth of the Δ*lsdD* mutant on DCA is complicated by SYK-6’s inability to grow without amino acid supplementation. Nevertheless, these results suggest that LSD4 is important for DCA catabolism in SYK-6. More generally, the genes encoding LSDs are distributed throughout the SYK-6 genome, suggesting that stilbenoids are intermediates in several pathways. Interestingly, *lsd7* is the homolog located closest to the proposed DCA catabolic genes (Fig. 1), lying within ∼5 kb of *phcCD* and *phcFGH*, respectively (14, 15, 16). However, LSD7 has relatively poor activity for DCA-S.

In conclusion, this study provides insight into the catabolic capabilities of the multiple LSD homologs of SYK-6. There appears to be considerable redundancy in the function of these enzymes, and further work is required to establish their physiological roles.

## Experimental procedures

### Chemicals and reagents

All reagents were of analytical grade unless otherwise noted. Restriction enzymes and the Phusion PCR system used for cloning were from New England Biolabs. Water for buffers was purified using a Barnstead Nanopure Diamond system to a resistance of at least 18 MΩ. Lignostilbene was a gift from Prof. Victor Snieckus and Dr Timothy E. Hurst (Queens University, Ontario). DCA-S was synthesized following an established protocol and described further in the Supporting information (15).

### DNA manipulation

DNA was purified, manipulated, and propagated using standard procedures (38). The *LSD* genes were amplified from genomic DNA prepared from SYK-6 (NBRC 103272) and the resulting amplicons were subcloned into pET41b (Novagen). The nucleotide sequences of the overexpression constructs were confirmed. The gene locus tags and the oligonucleotides used in this study are summarized in Table S1.

### Protein production and purification

The LSDs were produced heterologously using *E. coli* BL-21 λ(DE3) using the corresponding construct. The strains producing LSD1, LSD2, and LSD8 also contained pGro7 (Takara Bio Inc) for the production of GroEL and GroES chaperones. Freshly transformed cells were grown at 37 °C at 200 rpm in LB broth supplemented with 50 mg/l of kanamycin. The strains containing pGro7 were supplemented with 30 mg/l chloramphenicol and 1 mg/ml L-arabinose. Expression of the *LSD*s was induced with 1 mM isopropyl β-D-thiogalactopyranoside, at which time the medium was further supplemented with 0.5 mM FeCl_3_, and the cells were incubated at 30 °C for an additional 16 h prior to harvesting. Cells were harvested by centrifugation and stored at −80 °C until further processing.

The lysates for activity assay screening were prepared by suspending the cell pellets in 20 mM 4-(2-hydroxyethyl)-1-piperazinepropanesulfonic acid (HEPPS), pH 8.0. The suspended biomass was mixed with an equal volume of BugBuster HT solution (EMD Millipore); the mixture was incubated for 30 min under constant agitation at room temperature and cleared by centrifugation. The cleared lysates were kept at 4 °C and used the same day.

Protein for steady-state kinetic assays and crystallography was purified as follows. The frozen cells were thawed, suspended in 20 mM HEPPS, pH 8.0, and lysed at 4 °C using an EmulsiFlex-C5 homogenizer (Avestin). Cellular debris was removed by centrifugation. (NH_4_)_2_SO_4_ was added to the cleared lysate to a final concentration of 1.4 M, and the precipitate was removed by centrifugation. More (NH_4_)_2_SO_4_ was added to the supernatant to a final concentration of 2.2 M and protein was pelleted by centrifugation. The pellet was suspended with 20 mM HEPPS, 1.4 M (NH_4_)_2_SO_4_, pH 8.0, and the precipitate was removed by centrifugation and filtration at 0.45 μm. The supernatant was loaded onto an ÄKTA fast protein liquid chromatography system (GE Healthcare) and Source 15 phenyl column. The protein was eluted with a linear gradient from 1.4 to 0 M (NH_4_)_2_SO_4_ in 120 ml of 20 mM HEPPS, pH 8.0. Fractions containing LSD, as determined through SDS-PAGE, were pooled and dialyzed into 20 mM HEPPS, pH 8.0. LSD was purified further using a Source 15 Q column (GE Healthcare). The protein was eluted with a linear gradient from 0.2 to 1 M NaCl in 120 ml of 20 mM HEPPS, pH 8.0. Fractions containing LSD were pooled, dialyzed into 20 mM HEPPS, pH 8.0, concentrated to ∼20 mg/ml, flash-frozen as beads in liquid N_2_, and stored at −80 °C until further use.

### Protein analytical methods

Protein purity was evaluated using SDS–polyacrylamide gel stained with Coomassie Blue according to established procedures (38). Protein concentration was determined using a micro BCA Protein Assay Kit (Pierce) using bovine serum albumin as a standard. Iron concentrations were determined colorimetrically using a Ferene-S-based assay and ferric chloride solution as a standard (39). ICP-MS was performed using a NexION 300d (PerkinElmer) calibrated using IV-Stock-4 synthetic standard (Inorganic Ventures). To liberate metals ions, the protein samples were treated with concentrated HNO_3_ and H_2_O_2_ as previously described (40). For SEC-MALS analysis, LSD4 was dialyzed into 20 mM HEPES, pH 7.5, 100 mM NaCl, brought to 2 mg/ml, and loaded onto a Superdex 75 10/300 GL column (GE Healthcare) and operated at 0.4 ml/min at room temperature using the same buffer. Data were collected using a DAWN HELEOS II 16-angle light scattering detection module and an Optilab T-rEX differential refractometer (Wyatt Technologies). Data were analyzed using the ASTRA6 software (Wyatt Technologies).

### Steady-state kinetics

Kinetic assays were performed by monitoring the consumption of O_2_ using a Clark-type polarographic O_2_ electrode OXYG1 or OXYG1+ (Hansatech) connected to a circulating water bath. Assays were performed in 1 ml of air-saturated 40 mM HEPES (*I* = 0.1 M, pH 7.5) at 25 °C and initiated by adding the stilbenoid substrate. Stock solutions of the stilbenoids were made in dimethylformamide (DMF). The final concentration of DMF in the assay solutions was <0.5% (v/v). The electrode was calibrated daily according to the manufacturer’s instructions using air-saturated buffer and O_2_-depleted buffer *via* addition of sodium hydrosulfite. Stock solutions were prepared fresh daily. The steady-state kinetic parameters for O_2_ were assessed using 100 μM lignostilbene or DCA-S. Initial O_2_ concentration was varied using mixtures of buffers saturated with O_2_ or N_2_, respectively. The inhibition of LSD4 by vanillin was evaluated using air-saturated buffer and measuring the initial velocity as a function of lignostilbene and vanillin concentrations. Steady-state kinetic parameters were evaluated by fitting the Michaelis–Menten equation to the data using the least-squares fitting of LEONORA (41).

### Determination of reaction stoichiometry

Reaction stoichiometry was determined by correlating the amounts of O_2_ consumed and vanillin produced using an oxygraph and HPLC-based assays. The sample was prepared by reacting 50 μM lignostilbene or DCA-S with ∼0.1 μΜ LSD4 in air-saturated 40 mM HEPES (*I* = 0.1 M, pH 7.5) at 25 °C. The reaction was quenched upon the consumption of ∼50 μM O_2_ with 1% (v/v) glacial acetic acid. The protein was removed by centrifugation and passage through 0.22 μm filter. Vanillin produced from the LSD4-catalyzed reaction was quantified using an Agilent 1290 series UPLC system equipped with a Luna 2.5 um C18(2)-HST 100 Å column (Phenomenex) and a gradient of acetonitrile in 0.16% formic acid. Vanillin was detected at 265 nm using an Agilent 1290 diode array detector. Peak areas were converted to concentrations using a calibration curve established with authentic vanillin (R^2^ > 0.995).

### DCA-S cleavage products characterization

The sample was prepared by reacting ∼100 μM DCA-S, dissolved in DMF, with ∼1 μM LSD4 in air-saturated potassium phosphate pH 7.5; *I* = 0.1 M. The reaction was incubated with constant mixing at room temperature for ∼10 min prior to quenching with ∼10% (v/v) glacial acetic acid. The precipitated enzyme was removed by centrifugation and passaging through a 0.22 μm filter. The sample was resolved using a Waters 2695 Separation HPLC module equipped with a Waters 2996 photodiode array detector and a Luna C18(2) 5-μm 150 × 3-mm column (Phenomenex) with a linear gradient of 0.1% formic acid and methanol. DCA-S and vanillin were run as standards. GC-MS analyses were performed using an Agilent 8890 GC equipped with two HP-5 MS 15 m × 0.25 mm capillary columns fitted in series and a 5977B mass-selective detector. The sample was acidified with ∼5% (v/v) HCl, extracted using ethyl acetate, and dried. The dried sample was solubilized in an equal volume of pyridine and *N*,*O*-bis(trimethylsilyl)trifluoroacetamide: trimethyl-chlorosilane (99:1).

### Protein structure determination

Crystals of Fe-LSD4 were grown aerobically by sitting drop at room temperature in a 1:1 mixture of ∼40 mg/ml Fe-LSD4 in 20 mM HEPPS, pH 8.0 with reservoir solution containing 0.4 M magnesium acetate and 28% PEG3350 (w/v). Diffraction data were collected at the homesource facility with no additional cryoprotectant and processed using HKL3000 (42). Fe-LSD4 crystallized in space group *I*222 with a single protomer in its asymmetric unit. The structure was solved using molecular replacement with LsdA_TMY1009_ (PDB entry: 6OJR) as a search model and refined using Phenix (43, 44). The refined structure has residues 2 to 489 modeled for chain A, with residues 382 to 390 not modeled due to poor electron density.

Crystals of Co-LSD4 were grown aerobically by sitting drop at room temperature in a 1:2 or 1:3 mixture of ∼40 mg/ml Co-LSD4 in 20 mM HEPPS, pH 8.0 with reservoir solution containing 0.2 to 0.4 M magnesium acetate and 28 to 30% PEG3350 (w/v). Diffraction data were collected at the Canadian Light Source on beamline 08-ID. Data were processed using XDS (45). Co-LSD4 crystallized in the space group *I*222 with a single protomer in its asymmetric unit. The structure was solved using molecular replacement with LSD4 (PDB entry: 6XMA) as a search model in the program PhaserMR from the Phenix package (43, 44). The refined structure includes residues 2 to 489, with residues 385 to 388 not modeled due to poor electron density.

A crystal structure of Co-LSD4·vanillin was obtained by systematically replacing a drop solution containing Co-LSD4 crystals with well solution, followed by cooling of the crystal drop to 8 °C in a TG40-M device (Centeo Biosciences). Lignostilbene (200 mM prepared in DMSO) was diluted 1:10 into well solution, chilled to 8 °C, and added to the crystal drop to a final concentration of ∼10 mM. The crystal chamber was sealed and incubated overnight. Crystals were vitrified in liquid nitrogen and diffraction data were collected on a homesource facility and processed using HKL3000 (42). The structure was refined using Phenix (43, 44) and includes residues 2 to 489, with residues 382 to 390 not modeled due to poor electron density.

Crystal structures of Co-LSD4·lignostilbene and Co-LSD4·DCA-S were obtained by transfer of the crystals into an anaerobic glovebox chamber. Each substrate was dissolved into DMSO, diluted 1:10 into well solution and immediately mixed with the crystal-containing drop to a final concentration of ∼15 and 8 mM for lignostilbene and DCA-S, respectively. After incubation for ∼0.5 h (lignostilbene) and 1.5 h (DCA-S), the wells were sealed and removed from the glovebox. The crystals were immediately vitrified in liquid nitrogen. Diffraction data were collected at the Canadian Light Source on beamlines 08B1-1 and 08ID-1 and were processed using XDS (45). Both refined structures include residues 2 to 482, but residues 383 to 387 are not modeled in the lignostilbene structure due to poor electron density.

Data collection and refinement statistics for all five structures are summarized in Table S2. Structure figures were generated in PyMOL (Schrödinger, LLC). RMSD calculations between different structures were performed using the least-squared superposition tool of Coot or DALI (46, 47).

## Data availability

The structures presented in this paper have been deposited in the PDB with the following codes: 6XMA, 6XM6, 6XM7, 6XM8, and 6XM9 (Table S2). All other data are contained within the article.

## Supporting information

This article contains supporting information (15).

## Conflict of interest

The authors declare that they have no conflicts of interest with the contents of this article.

## References

[1] Eltis L.D., Singh R. (2018). Chapter 11 biological funneling as a means of transforming lignin-derived aromatic compounds into value-added chemicals. Lignin Valorization: Emerging Approaches.

[2] Becker J., Wittmann C. (2019). A field of dreams: Lignin valorization into chemicals, materials, fuels, and health-care products. Biotechnol. Adv..

[3] Beckham G.T., Johnson C.W., Karp E.M., Salvachúa D., Vardon D.R. (2016). Opportunities and challenges in biological lignin valorization. Curr. Opin. Biotechnol..

[4] Kamimura N., Takahashi K., Mori K., Araki T., Fujita M., Higuchi Y., Masai E. (2017). Bacterial catabolism of lignin-derived aromatics: New findings in a recent decade: Update on bacterial lignin catabolism. Environ. Microbiol. Rep..

[5] Seaton S.C., Neidle E.L. (2018). Chapter 10 using aerobic pathways for aromatic compound degradation to engineer lignin metabolism. Lignin Valorization: Emerging Approaches.

[6] Ragauskas A.J., Beckham G.T., Biddy M.J., Chandra R., Chen F., Davis M.F., Davison B.H., Dixon R.A., Gilna P., Keller M., Langan P., Naskar A.K., Saddler J.N., Tschaplinski T.J., Tuskan G.A. (2014). Lignin valorization: Improving lignin processing in the biorefinery. Science.

[7] Schutyser W., Renders T., Van den Bosch S., Koelewijn S.-F., Beckham G.T., Sels B.F. (2018). Chemicals from lignin: An interplay of lignocellulose fractionation, depolymerisation, and upgrading. Chem. Soc. Rev..

[8] Johnson C.W., Salvachúa D., Rorrer N.A., Black B.A., Vardon D.R., St. John P.C., Cleveland N.S., Dominick G., Elmore J.R., Grundl N., Khanna P., Martinez C.R., Michener W.E., Peterson D.J., Ramirez K.J. (2019). Innovative chemicals and materials from bacterial aromatic catabolic pathways. Joule.

[9] Katayama Y., Nishikawa S., Nakamura M., Yano K., Yamasaki M., Morohoshi N., Haraguchi T. (1987). Cloning and expression of *Pseudomonas paucimobilis* SYK-6 genes involved in the degradation of vanillate and protocatechuate in *P. putida*. Mokuzai Gakkaishi.

[10] Harada A., Kamimura N., Takeuchi K., Yu H.Y., Masai E., Senda T. (2017). The crystal structure of a new *O*-demethylase from *Sphingobium* sp. strain SYK-6. FEBS J..

[11] Varman A.M., He L., Follenfant R., Wu W., Wemmer S., Wrobel S.A., Tang Y.J., Singh S. (2016). Decoding how a soil bacterium extracts building blocks and metabolic energy from ligninolysis provides road map for lignin valorization. Proc. Natl. Acad. Sci. U. S. A..

[12] Sonoki T., Takahashi K., Sugita H., Hatamura M., Azuma Y., Sato T., Suzuki S., Kamimura N., Masai E. (2018). Glucose-free *cis,cis*-muconic acid production via new metabolic designs corresponding to the heterogeneity of lignin. ACS Sustain. Chem. Eng..

[13] Suzuki Y., Okamura-Abe Y., Nakamura M., Otsuka Y., Araki T., Otsuka H., Navarro R.R., Kamimura N., Masai E., Katayama Y. (2020). Development of the production of 2-pyrone-4,6-dicarboxylic acid from lignin extracts, which are industrially formed as by-products, as raw materials. J. Biosci. Bioeng..

[14] Takahashi K., Kamimura N., Hishiyama S., Hara H., Kasai D., Katayama Y., Fukuda M., Kajita S., Masai E. (2014). Characterization of the catabolic pathway for a phenylcoumaran-type lignin-derived biaryl in *Sphingobium* sp. strain SYK-6. Biodegradation.

[15] Takahashi K., Hirose Y., Kamimura N., Hishiyama S., Hara H., Araki T., Kasai D., Kajita S., Katayama Y., Fukuda M., Masai E. (2015). Membrane-associated glucose-methanol-choline oxidoreductase family enzymes PhcC and PhcD are essential for enantioselective catabolism of dehydrodiconiferyl alcohol. Appl. Environ. Microbiol..

[16] Takahashi K., Miyake K., Hishiyama S., Kamimura N., Masai E. (2018). Two novel decarboxylase genes play a key role in the stereospecific catabolism of dehydrodiconiferyl alcohol in *Sphingobium* sp. strain SYK-6. Environ. Microbiol..

[17] Kamoda S., Terada T., Saburi Y. (2003). A common structure of substrate shared by lignostilbene dioxygenase isozymes from *Sphingomonas paucimobilis* TMY1009. Biosci. Biotechnol. Biochem..

[18] Kamoda S., Habu N., Samejima M., Yoshimoto T. (1989). Purification and some properties of lignostilbene-α-β-dioxygenase responsible for the C-α-C-β cleavage of a diarylpropane type lignin model-compound from *Pseudomonas* sp. TMY1009. Agric. Biol. Chem..

[19] Kamoda S., Saburi Y. (1993). Structural and enzymatical comparison of lignostilbene-α,β-dioxygenase isozymes, I, II, and III, from *Pseudomonas paucimobilis* TMY1009. Biosci. Biotechnol. Biochem..

[20] Daruwalla A., Kiser P.D. (2019). Structural and mechanistic aspects of carotenoid cleavage dioxygenases (CCDs). Biochim. Biophys. Acta Mol. Cell Biol. Lipids.

[21] Sui X., Kiser P.D., Lintig J., Palczewski K. (2013). Structural basis of carotenoid cleavage: From bacteria to mammals. Arch. Biochem. Biophys..

[22] Harrison P.J., Bugg T.D. (2014). Enzymology of the carotenoid cleavage dioxygenases: Reaction mechanisms, inhibition and biochemical roles. Arch. Biochem. Biophys..

[23] Sui X., Zhang J., Golczak M., Palczewski K., Kiser P.D. (2016). Key residues for catalytic function and metal coordination in a carotenoid cleavage dioxygenase. J. Biol. Chem..

[24] McAndrew R.P., Sathitsuksanoh N., Mbughuni M.M., Heins R.A., Pereira J.H., George A., Sale K.L., Fox B.G., Simmons B.A., Adams P.D. (2016). Structure and mechanism of NOV1, a resveratrol-cleaving dioxygenase. Proc. Natl. Acad. Sci. U. S. A..

[25] Kishi K., Habu N., Samejima M., Yoshimoto T. (1991). Purification and some properties of the enzyme catalyzing the C-γ-elimination of a diarylpropane-type lignin model from *Pseudomonas paucimobilis* TMY1009. Agric. Biol. Chem..

[26] Presley G.N., Werner A.Z., Katahira R., Garcia D.C., Haugen S.J., Ramirez K.J., Giannone R.J., Beckham G.T., Michener J.K. (2021). Pathway discovery and engineering for cleavage of a β-1 lignin-derived biaryl compound. Metab. Eng..

[27] Kamoda S., Terada T., Saburi Y. (1997). Purification and some properties of lignostilbene-α,β-dioxygenase isozyme IV from *Pseudomonas paucimobilis* TMY1009. Biosci. Biotechnol. Biochem..

[28] Kurt Z., Minoia M., Spain J.C. (2018). Resveratrol as a growth substrate for bacteria from the rhizosphere. Appl. Environ. Microbiol..

[29] Yu R.Q., Kurt Z., He F., Spain J.C. (2019). Biodegradation of the allelopathic chemical pterostilbene by a *Sphingobium* sp. strain from the peanut rhizosphere. Appl. Environ. Microbiol..

[30] Brefort T., Scherzinger D., Limon M.C., Estrada A.F., Trautmann D., Mengel C., Avalos J., Al-Babili S. (2011). Cleavage of resveratrol in fungi: Characterization of the enzyme Rco1 from Ustilago maydis. Fungal Genet. Biol..

[31] Sui X., Farquhar E.R., Hill H.E., von Lintig J., Shi W., Kiser P.D. (2018). Preparation and characterization of metal-substituted carotenoid cleavage oxygenases. J. Biol. Inorg. Chem..

[32] Sui X., Weitz A.C., Farquhar E.R., Badiee M., Banerjee S., von Lintig J., Tochtrop G.P., Palczewski K., Hendrich M.P., Kiser P.D. (2017). Structure and spectroscopy of alkene-cleaving dioxygenases containing an atypically coordinated non-heme iron center. Biochemistry.

[33] Kuatsjah E., Verstraete M.M., Kobylarz M.J., Liu A.K.N., Murphy M.E.P., Eltis L.D. (2019). Identification of functionally important residues and structural features in a bacterial lignostilbene dioxygenase. J. Biol. Chem..

[34] Kamimura N., Hirose Y., Masuba R., Kato R., Takahashi K., Higuchi Y., Hishiyama S., Masai E. (2021). LsdD has a critical role in the dehydrodiconiferyl alcohol catabolism among eight lignostilbene-α,β-dioxygenase isozymes in *Sphingobium* sp. strain SYK-6. Int. Biodeter. Biodegr..

[35] Khadka N., Farquhar E.R., Hill H.E., Shi W., von Lintig J., Kiser P.D. (2019). Evidence for distinct rate-limiting steps in the cleavage of alkenes by carotenoid cleavage dioxygenases. J. Biol. Chem..

[36] Yamada M., Okada Y., Yoshida T., Nagasawa T. (2007). Purification, characterization and gene cloning of isoeugenol-degrading enzyme from *Pseudomonas putida* IE27. Arch. Microbiol..

[37] Kiser P.D., Farquhar E.R., Shi W., Sui X., Chance M.R., Palczewski K. (2012). Structure of RPE65 isomerase in a lipidic matrix reveals roles for phospholipids and iron in catalysis. Proc. Natl. Acad. Sci. U. S. A..

[38] Ausubel F.M. (2002). Short Protocols in Molecular Biology: A Compendium of Methods from Current Protocols in Molecular Biology.

[39] Haigler B.E., Gibson D.T. (1990). Purification and properties of NADH-ferredoxin NAP reductase, a component of naphthalene dioxygenase from *Pseudomonas* sp. strain NCIB 9816. J. Bacteriol..

[40] Shiel A.E., Weis D., Orians K.J. (2012). Tracing cadmium, zinc and lead sources in bivalves from the coasts of western Canada and the USA using isotopes. Geochim. Cosmochim. Acta.

[41] Cornish-Bowden A. (1995). Analysis of Enzyme Kinetic Data.

[42] Minor W., Cymborowski M., Otwinowski Z., Chruszcz M. (2006). HKL-3000: The integration of data reduction and structure solution--from diffraction images to an initial model in minutes. Acta Crystallogr. D Biol. Crystallogr..

[43] McCoy A.J., Grosse-Kunstleve R.W., Adams P.D., Winn M.D., Storoni L.C., Read R.J. (2007). Phaser crystallographic software. J. Appl. Crystallogr..

[44] Adams P.D., Afonine P.V., Bunkoczi G., Chen V.B., Davis I.W., Echols N., Headd J.J., Hung L.W., Kapral G.J., Grosse-Kunstleve R.W., McCoy A.J., Moriarty N.W., Oeffner R., Read R.J., Richardson D.C. (2010). PHENIX: A comprehensive Python-based system for macromolecular structure solution. Acta Crystallogr. D Struct. Biol..

[45] Kabsch W. (2010). XDS. Acta Crystallogr. D Biol. Crystallogr..

[46] Holm L., Sander C. (1995). Dali: A network tool for protein structure comparison. Trends Biochem. Sci..

[47] Emsley P., Lohkamp B., Scott W.G., Cowtan K. (2010). Features and development of Coot. Acta Crystallogr. D Struct. Biol..

