# Supplementary material for: Structural and functional analysis of lignostilbene dioxygenases from Sphingobium sp. SYK-6
Source: J Biol Chem. 2021 May 7;296:100758. doi: 10.1016/j.jbc.2021.100758 (PMC8191317; doi:10.1016/j.jbc.2021.100758)
Supplement: Supplemental Figures S1–S9 and Tables S1–S4 [file mmc1.pdf]

SUPPLEMENTAL MATERIALS

DCA-S synthesis

DCA-S was prepared from vanillin and 1-bromo-5-formylguaiacol through 8 steps according to an established protocol (1). The synthetic route is shown in Scheme **1**. All chemicals were purchased from commercial sources and used without additional purification. In a standard post-treatment procedure, reaction mixtures were extracted with ethyl acetate three times. Combined ethyl acetate layers were washed with brine and dried over Na_2SO_4. After filtration, filtrates were concentrated using a rotary evaporator. NMR spectra were acquired on a Bruker 400 MHz NMR spectrometer with a 5 mm BBO probe with a Z gradient at 25°C. 1D (proton and carbon) and 2D-NMR spectra gradient correlation COSY, heteronuclear single quantum coherence were recorded using the standard Bruker implementation. The TMS and solvent peaks were used as an internal reference (δ_H/δ_C 0/0 ppm for TMS and δ_H/δ_C 2.5/77.23 ppm for $CDCl_3$).

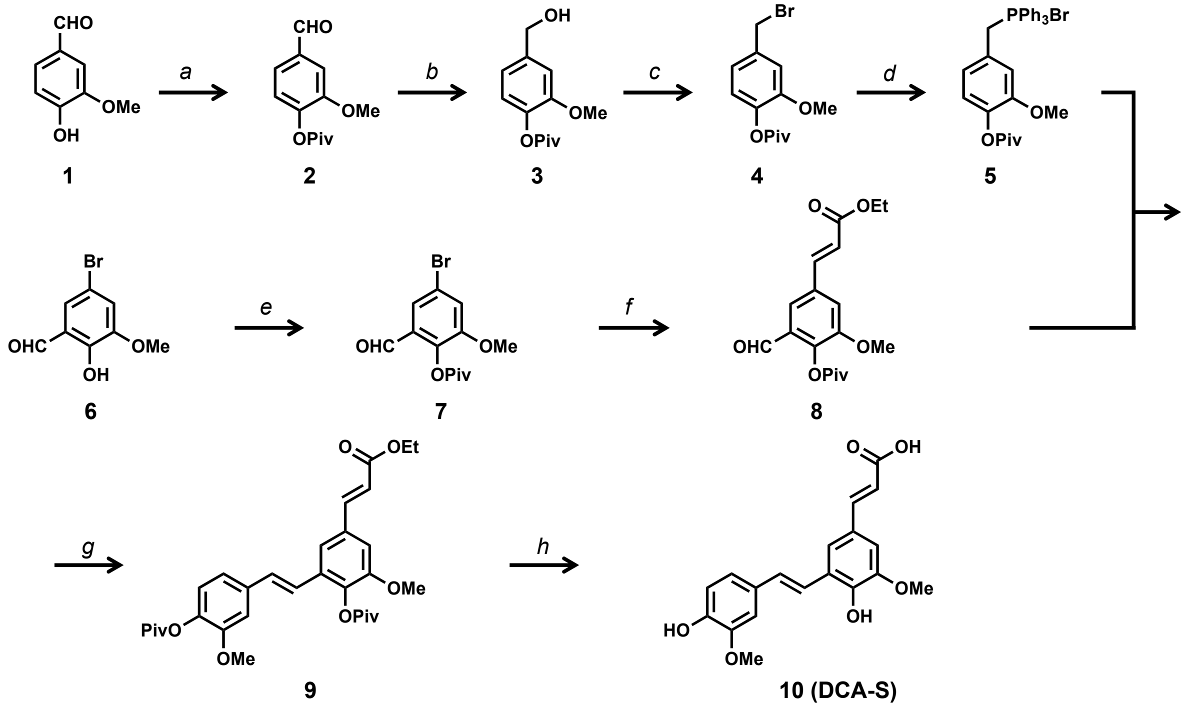

[a] PivCl, pyridine, CH_2Cl_2, 0 ˚C→rt, 12h [b] NaBH4, MeOH, rt, 1h [c] NBS, PPh_3, THF, rt, 1.5h [d] PPh_3, benzene, reflux, 4h [e] PivCl, pyridine, CH_2Cl_2, 0˚C→rt, 12h [f] Ethyl acrylate, Et_3N, $Pd(OAc)_2$, PPh_3, DMF, 90 ˚C, 6h, [g] ^{t}BuOK, 0 ˚C→rt, 5h, [h] 2N-NaOH, dioxane, rt, overnight

Scheme 1. Synthetic route of DCA-S

Synthesis of compound 3

To a solution of vanillin **1** (12 g, 78.9 mM) in anhydrous dichloromethane (DCM, 150 ml) and pyridine (13.4 ml), pivaloyl chloride (13.6 ml, 110.4 mM) was added dropwise over 20 min at 0 °C. After stirring for 10 min, the reaction temperature increase to room temperature. The reaction was finished after 5.5 h. Saturated NH_4Cl solution was added to the reaction mixture, followed by the standard post-treatment. The obtained

pivaloylate **2** was used for the next reduction without purification. To the solution of compound **2** in MeOH (100 ml), $NaBH_4$ (8.95 g, 236.7 mM) was added stepwise at 0°C. After stirring for 10 min, the reaction temperature was increased to room temperature. After stirring for 2 h, the reaction mixture was diluted with saturated NH_4Cl solution, followed by the standard post-treatment. The crude product was purified by silica gel chromatography with ethyl acetate /Hexane (1:2, v/v) to give compound **3** with 60.8% yield. ^{1}H NMR(400 MHz, $CDCl_3$) δ 6.96 (1H, d, *J* = 1.2 Hz), 6.94 (1H, d, *J* = 8.2 Hz), 6.86 (1H, d, *J* = 1.2 Hz), 4.62 (2H, s, CH_2), 3.78 (3H, s, OMe), 1.35 (9H, s, (-C($\underline{C}H_3)_3$). ^{13}C NMR (100 MHz, $CDCl_3$) δ, 176.9 (-$\underline{C}OC(CH_3)_3$), 151.3, 139.6, 139.5, 122.6, 118.9, 111.1, 65.1 (Cα), 56.1 (OMe), 39.1 (-$\underline{C}(CH_3)_3$), 27.2 (-C($\underline{C}H_3)_3$) ppm

Synthesis of compound 4

To a solution of compound **3** (2.83 g, 11.9 mM) in anhydrous THF (25 ml), triphenylphosphine (3.27 g, 12.5 mM) and N-bromosuccinimide (2.24 g, 12.5 mM) were added at 0°C. After stirring for 4 h at the same temperature, the solvent in the reaction mixture was removed by a rotary evaporator. The crude mixture was purified by silica gel chromatography with ethyl acetate / hexane (1:4, v/v) to give compound **4** (2.72 g) with 76.2% yield. ^{1}H NMR (400 MHz, $CDCl_3$) δ 6.97-6.95 (3H, m), 4.46 (1H, s, Hα), 3.80 (3H, s, OMe), 1.35 (9H, s, $C(CH_3)_3$). ^{13}C NMR (100 MHz, $CDCl_3$) δ 176.7 (-$\underline{C}OC(CH_3)_3$), 151.5, 140.6, 136.4, 123.1, 121.5, 113.4, 56.1 (OMe), 39.3 (-$\underline{C}(CH_3)_3$), 33.5 (Cα), 27.4 (-C($\underline{C}H_3)_3$) ppm.

Synthesis of compound 5

To a solution of compound **4** (2.72 g, 9.04 mM) in anhydrous benzene (40 ml), triphenylphosphine (2.49 g, 9.45 mM) was added at room temperature. After refluxing for 12 h, the reaction mixture was filtered to obtain phosphonium salt **5** as a white powder (3.43 g) with 69.0 % yield. ^{1}H NMR (400 MHz, $CDCl_3$) δ 7.78-7.60 (15H, m), 7.07 (1H, t, *J* = 2.0 Hz), 6.68 (1H, dd, *J* = 8.1, 0.52Hz), 6.52 (1H, dt, *J* = 8.0, 2.34 Hz), 5.20 (2H, d, *J* = 14.2 Hz), 3.46 (3H, s, OMe), 1.33 (9H, s, $C(CH_3)_3$). ^{13}C NMR (100 MHz, $CDCl_3$) δ 177.0 (-$\underline{C}OC(CH_3)_3$), 151.2, 140.2, 135.0, 134.7, 134.6, 130.2, 130.1, 126.0, 125.9, 123.53, 123.47, 122.7,118.1, 117.3, 116.8, 56.4 (OMe), 39.1 (-$\underline{C}(CH_3)_3$), 30.2, 29.8, 27.3 (-C($\underline{C}H_3)_3$) ppm.

Synthesis of compound 7

To a solution of 5-bromo-2-hydroxy-3-methoxybenzaldehyde **6** (1 g, 4.21 mM) in anhydrous DCM (7 ml) and pyridine (0.61 ml), pivaloyl chloride (0.62 ml, 5.05 mM) was added stepwise over 30 min at 0°C. After stirring for 10 min, the reaction temperature was increased to room temperature. After stirring for 10 h, a saturated NH_4Cl solution was added to the reaction mixture and extracted with DCM 3 times. The combined DCM layer was washed with brine and then dried over Na_2SO_4. The crude product was purified by silica gel chromatography with ethyl acetate / hexane (1:4, v/v) to give compound **7** with 96.3% yield. ^{1}H NMR (400 MHz, $CDCl_3$) δ 10.0 (1H, s, CHO), 7.49 (1H, d, *J* = 2.2 Hz, H6), 7.25 (1H, d, *J* = 2.1 Hz, H2), 3.78 (3H, s, OMe), 1.39 (9H, s, $C(CH_3)_3$). ^{13}C NMR (100 MHz, $CDCl_3$) δ 186.2 (1H, s, CHO), 175.3 (-$\underline{C}OC(CH_3)_3$), 152.3, 141.6, 129.9, 122.1, 120.4, 119.1, 56.2 (OMe), 39.0 (-$\underline{C}(CH_3)_3$), 26.7 (-C($\underline{C}H_3)_3$) ppm.

Synthesis of compound 8

To a solution of the pivaloylate **7** (5.0 g, 17.4 mM) in DMF (15 ml), ethyl acrylate (17.7 ml, 160 mM), triethylamine (3.66 ml, 126.1 mM), palladium (II) acetate (78.2 mg, 0.348 mM) and triphenylphosphine (183 mg, 0.696 mM) were added at 90°C. After stirring for 26 h, the reaction mixture was diluted with d.i-H_2O (50 ml) and then the standard post-treatment was conducted. The crude product was purified by silica gel chromatography with ethyl acetate / hexane (1:2, v/v) to give compound **7** (1.7 g, 30.3% yield). ^{1}H NMR (400

MHz, $CDCl_3$): δ 10.1 (1H, s, CHO), 7.65 (1H, d, *J* = 16.0 Hz), 7.62 (1H, d, *J* = 1.92 Hz), 7.29 (1H, d, *J* = 1.92 Hz), 6.45 (1H, d, *J* = 16.0 Hz), 4.28 (2H, q, *J* = 7.2 Hz, -C$\underline{H}_2$$CH_3$), 3.88 (3H, s, OMe), 1.42 (9H, s, $C(CH_3)_3$), 1.34 (3H, t, *J* = 7.12 Hz, -CH_2C$\underline{H}_3$). ^{13}C NMR (100 MHz, $CDCl_3$) δ 187.9 (1H, s, CHO), 176.1 (-$\underline{C}$OC$(CH_3)_3$), 166.6, 152.4, 144.3, 142.9, 133.4, 130.0, 120.3, 120.2, 116.0, 60.9 (-$\underline{C}H_2CH_3$), 56.7 (OMe), 39.6 (-$\underline{C}(CH_3)_3$), 27.3 (-C$(\underline{C}H_3)_3$), 14.5 (-$CH_2\underline{C}H_3$) ppm.

Synthesis of compound 9

To a solution of pivaloylate **8** (3.0 g, 4.21 mM) and the phosphonium salt **5** (2.78 g, 5.05 mM) in THF, sodium tert-butoxide (0.485 g, 5.05 mM) was added at 0°C. After stirring for 7 h at room temperature, the reaction mixture was filtered to remove salt. After removal of solvent from the filtrate, the obtained crude product was purified by silica gel chromatography with ethyl acetate / hexane (1:4, v/v) to give compound **9** (0.6 g, 30.5% yield) as a syrup. ^{1}H NMR (400 MHz, $CDCl_3$) δ 7.67 (1H, d, *J* = 16 Hz, -CH-), 7.42 (1H, d, *J* = 1.6 Hz, -CH-), 7.11-6.97 (5H, m), 6.43 (1H, d, *J* = 15.9 Hz, -CH-), 4.28 (2H, dd, *J* = 14, 7.2 Hz, -C$\underline{H}_2$$CH_3$), 3.84 (3H, s, OMe), 3.83 (3H, s, OMe), 1.43 (9H, s, $C(CH_3)_3$), 1.37 (9H, s, $C(CH_3)_3$), 1.35 (3H, t, *J*=7.2Hz, -CH_2C$\underline{H}_3$). ^{13}C NMR (100 MHz, $CDCl_3$) δ 176.7 (-$\underline{C}$OC$(CH_3)_3$), 175.9 (-$\underline{C}$OC$(CH_3)_3$, 166.9 ($\underline{C}$OOEt), 152.0, 151.4, 144.2, 140.3, 139.5, 135.8, 132.6, 131.4, 131.1, 123.0, 121.0, 119.5, 118.5, 118.4, 110.1, 109.6, 60.6 (-$\underline{C}H_2CH_3$), 56.1 (OMe), 55.8 (OMe), 39.4 (-$\underline{C}(CH_3)_3$), 39.2 (-$\underline{C}(CH_3)_3$), 27.3 (-C$(\underline{C}H_3)_3$), 14.4 (-$CH_2\underline{C}H_3$) ppm.

Synthesis of compound 10 (DCA-S)

To a solution of compound **9** (0.11 g, 0.204 mM) in 1,4-dioxane (4 ml), 2 N NaOH (4 mM) was added dropwise at 0°C. After stirring for 1 h at the same temperature, the reaction mixture was diluted with d.i-H_2O (10 ml) and acidified with 1 N HCl, and then the standard post-treatment approach was conducted. The crude product was purified by preparative TLC with ethyl acetate / hexane (1:2, v/v) to give DCA-S **10** (0.07 g, 99% yield) as a pale-yellow powder. ^{1}H NMR (400 MHz, Acetone-d_6) δ 8.34 (1H, bs), 7.78 (1H, bs), 7.66 (1H, d, *J* = 15.9Hz), 7.56 (1H, d, *J* = 1.8 Hz), 7.34 (1H, S), 7.33 (1H, s), 7.25 (1H, d, *J* = 1.8 Hz), 7.24 (1H, d, *J* = 1.9 Hz), 7.07 (1H, dd, *J* = 8.2, 1.9 Hz), 6.85 (1H, d, *J* = 8.2 Hz), 6.46 (1H, d, *J* = 15.9 Hz), 3.96 (3H, s, OMe), 3.92 (3H, s, OMe). ^{13}C NMR (100 MHz, Acetone-d_6) δ 168.4, 148.9, 148.7, 147.7, 147.0, 146.2, 131.0, 130.7, 127.0, 125.5, 121.2, 120.9, 120.6, 116.5, 116.1, 110.2, 109.2, 56.6, 56.3 ppm

Table S1. Metal-ligand distances and ligand-metal-ligand angles for LSD4 and Co-LSD4 complexes

	LSD4		Co-LSD4		Co-LSD4 • DCA-S		Co-LSD4 • lignostilbene		Co-LSD4 • vanillin	
Distances (Å)	Fe - $N\varepsilon_2$ His167	2.25	Fe - $N\varepsilon_2$ His167	2.12	Fe - $N\varepsilon_2$ His167	2.05	Fe - $N\varepsilon_2$ His167	2.06	Fe - $N\varepsilon_2$ His167	2.12
	Fe - $N\varepsilon_2$ His218	2.00	Fe - $N\varepsilon_2$ His218	2.01	Fe - $N\varepsilon_2$ His218	1.98	Fe - $N\varepsilon_2$ His218	2.07	Fe - $N\varepsilon_2$ His218	2.08
	Fe - $N\varepsilon_2$ His284	2.08	Fe - $N\varepsilon_2$ His284	2.05	Fe - $N\varepsilon_2$ His284	2.01	Fe - $N\varepsilon_2$ His284	2.03	Fe - $N\varepsilon_2$ His284	2.01
	Fe - $N\varepsilon_2$ His476	2.06	Fe - $N\varepsilon_2$ His476	2.02	Fe - $N\varepsilon_2$ His476	2.02	Fe - $N\varepsilon_2$ His476	2.01	Fe - $N\varepsilon_2$ His476	2.03
	Fe - O Water449	2.26	Fe - O Water514	2.17	Fe - O Water543	2.26	Fe - O Water653	2.19	Fe - O Water557	2.16
Angles (degrees)	Water449 - Fe - His218	90.26	Water514 - Fe - His218	86.00	Water543 - Fe - His218	83.06	Water653 - Fe - His218	78.38	Water557 - Fe - His218	86.98
	Water449 - Fe - His284	88.33	Water514 - Fe - His284	92.59	Water543 - Fe - His284	93.55	Water653 - Fe - His284	92.06	Water557 - Fe - His284	102.79
	Water449 - Fe - His476	88.36	Water514 - Fe - His476	87.97	Water543 - Fe - His476	83.11	Water653 - Fe - His476	82.95	Water557 - Fe - His476	83.84
	His167 - Fe - His218	89.48	His167 - Fe - His218	91.76	His167 - Fe - His218	95.50	His167 - Fe - His218	106.79	His167 - Fe - His218	92.13
	His167 - Fe - His284	97.66	His167 - Fe - His284	99.05	His167 - Fe - His284	100.31	His167 - Fe - His284	104.41	His167 - Fe - His284	100.49
	His167 - Fe - His476	89.55	His167 - Fe - His476	89.25	His167 - Fe - His476	91.40	His167 - Fe - His476	94.13	His167 - Fe - His476	88.30
	His476 - Fe - His284	101.38	His476 - Fe - His284	100.78	His476 - Fe - His284	100.63	His476 - Fe - His284	102.39	His476 - Fe - His284	99.49
	His284 - Fe - His218	101.24	His284 - Fe - His218	103.92	His284 - Fe - His218	107.21	His284 - Fe - His218	104.84	His284 - Fe - His218	102.28

Table S2. X-ray diffraction data collection and refinement statistics for Fe and Co-LSD4 structures. [a]

	Fe-LSD4	Co-LSD4	Co-LSD4 • DCA-S	Co-LSD4 • lignostilbene	Co-LSD4 • vanillin
Data Collection[a]					
Resolution Range (Å)	50.00-1.45 (1.48-1.45)	50.00-1.45 (1.55-1.45)	50.00-1.45 (1.55-1.45)	50.00-1.85 (1.96-1.85)	50.00-1.65 (1.68-1.65)
Space group	*I222*	*I222*	*I222*	*I222*	*I222*
Unit cell dimensions(Å)	86.03 112.53 115.38 90 90 90	85.48 112.18 114.74 90 90 90	85.32 112.02 114.67 90 90 90	85.70 112.54 115.52 90 90 90	85.16 112.16 115.31 90 90 90
Unique reflections	98790	97483	95307	47940	66376
Completeness (%)	99.9 (97.3)	99.9 (99.6)	97.8 (88.9)	100.0 (100.0)	100.0 (99.7)
Redundancy	16.1	3.3	3.2	3.8	7.8
Average $I/\sigma I$	26.5 (2.0)	14.0 (3.0)	15.7 (1.9)	6.7 (2.1)	15.1 (1.8)
R_{merge}	0.11 (0.56)	0.05 (0.38)	0.04 (0.50)	0.15 (0.73)	0.12 (0.58)
$CC_{1/2}$ (highest res. shell)	0.829	0.921	0.854	0.708	0.808
Refinement					
R_{work} (R_{free})	0.149 (0.171)	0.139 (0.159)	0.134 (0.154)	0.147 (0.173)	0.159 (0.187)
Number of water molecules	885	798	679	683	706
RMSD bond length (Å)	0.008	0.012	0.013	0.005	0.008
RMSD bond angles (º)	1.071	1.237	1.288	0.779	1.000
Average *B*-value (Å^2)	16.6	19.7	21.8	19.7	17.4
Ramachandran Plot (%)					
Most favored regions	97.7	98.1	97.9	97.7	97.7
Disallowed regions	0	0	0	0	0
PDB entry	6XMA	6XM6	6XM7	6XM8	6XM9

** Highest resolution shell in brackets*

Table S3. Dimer interface analysis of structurally characterized LSDs using PDBePISA.

Protein	Interface area (Å^2)	H-bond	Salt-bridges
CAO1	1429	31	12
$LsdA_{TMY1009}$	1462	14	9
LSD_{Pbra}	1412	15	3
LSD4	992	-	-
LSD_{NOV1}	928	10	2

Table S4. Oligonucleotides used in this study

Oligonucleotide name	Nucleotide sequence[a]	Restriction site
Cloning		
*LSD1*_SLG_37540_F	CCCCATATGGCCCATTTCCCGCAGAC	NdeI
*LSD1*_SLG_37540_R	CCCAAGCTTTCAGGCGGCGACGGC	HindIII
*LSD2*_SLG_36640_F	CCCCATATGGCCCACTTCCCCGACAC	NdeI
*LSD2*_SLG_36640_R	CCCAAGCTTTCAGGCGGCGATGGGCA	HindIII
*LSD3*_SLG_11300_F	CCCCATATGACCACGTTTCCCGACGT	NdeI
*LSD3*_SLG_11300_R	CCCAAGCTTTCAGGCCGCCCGTTCC	HindIII
*LSD4*_SLG_12860_F	CCCCATATGGCACATTTTCCCGACAC	NdeI
*LSD4*_SLG_12860_R	CCCAAGCTTTCAGGCGGCGAGGCC	HindIII
*LSD5*_SLG_27970_F	CCCCATATGGCGAGTTTTCCCGACAC	NdeI
*LSD5*_SLG_27970_R	CCCAAGCTTCTACGCCGCCGCAGCG	HindIII
*LSD6*_SLG_12580_F	CCCCATATGTCATTTCCCGCGACCCC	NdeI
*LSD6*_SLG_12580_R	CCCAAGCTTCTAGGCCGCCCGCGCC	HindIII
*LSD7*_SLG_09440_F	CCCCATATGGTCGAACCAATCCACTT	NdeI
*LSD7*_SLG_09440_R	CCCAAGCTTTCAGGCCGCCGAGCT	HindIII
*LSD8*_SLG_27300_F	CCCCATATGTCCCAAACATCAGACTT	NdeI
*LSD8*_SLG_27300_R	CCCAAGCTTTCACCAGACATTGAGTTGC	HindIII
Mutagenesis[b]		
*LSD4*_S283A	/5PHOS/-GAGAACTGCTTCGCC**GC**CCATGTGATGAATG	
*LSD4*_S283F	/5PHOS/-GAGAACTGCTTCGCC**TT**CCATGTGATGAATGCC	

[a]Introduced restriction site underlined

[a]Mismatched nucleotides are denoted in bold

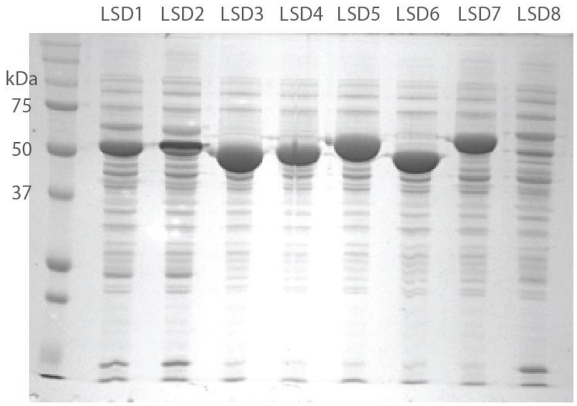

Figure S1. Production of soluble SYK-6 LSDs in *E. coli*.
SDS-PAGE loaded with cleared lysate of *E. coli* BL21(DE3) strains producing indicated LSD from SYK-6. Lane at far left loaded with Precision Plus Protein standards from Biorad. Molecular weight of three most relevant standards indicated.

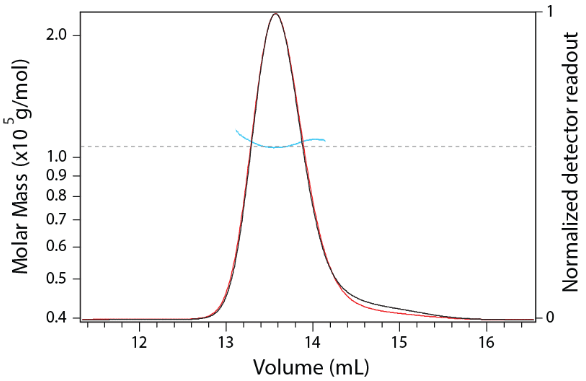

Figure S2. SEC-MALS analysis of Co-LSD4.
The black and red traces represent absorbance at 280 nm and light-scattering, respectively. The molar mass fit is shown in cyan.

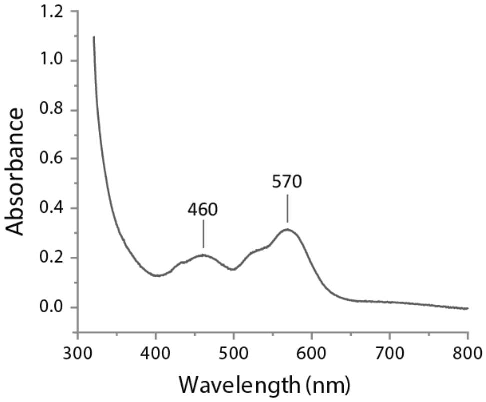

Figure S3. UV-vis absorbance spectrum of Co-LSD4.
Co-LSD4 was in HEPES (I = 0.1 M; pH: 7.5).

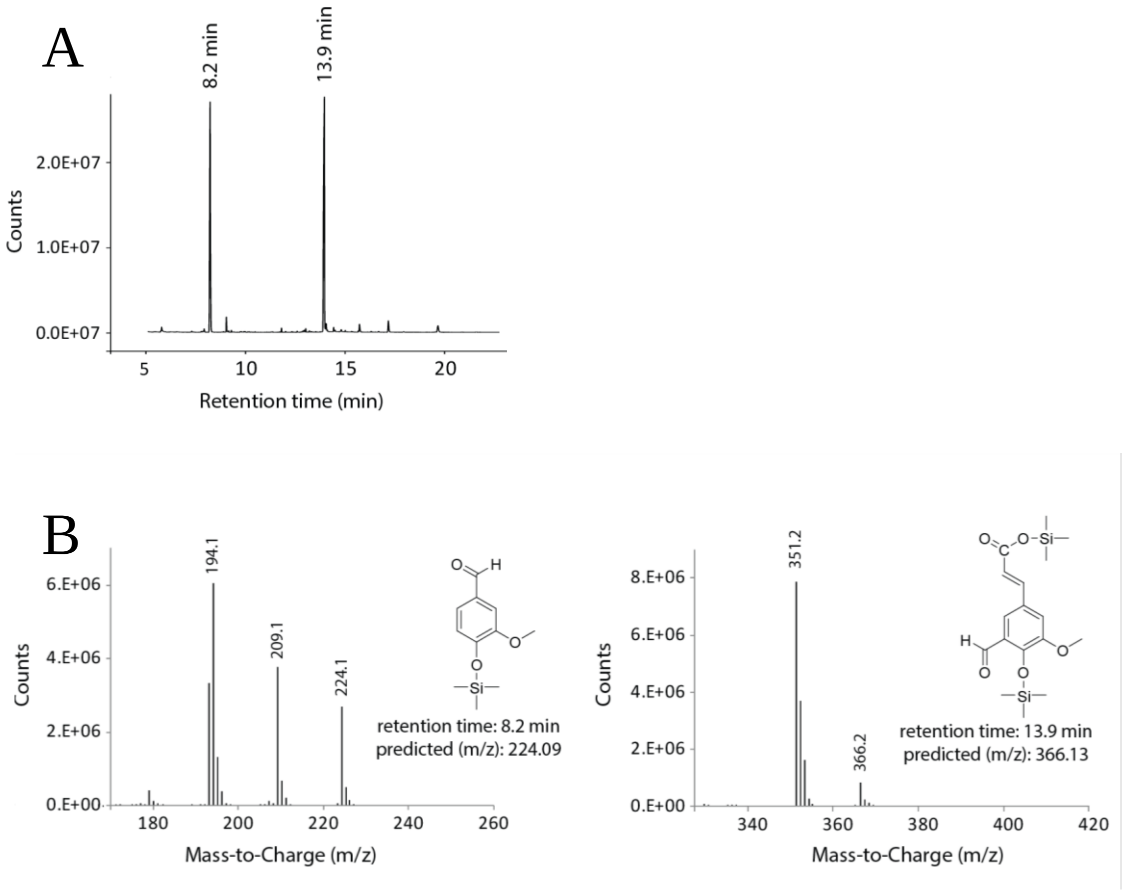

Figure S4. GC-MS resolution (A) and mass spectra (B) of LSD4-catalyzed DCA-S cleavage products.

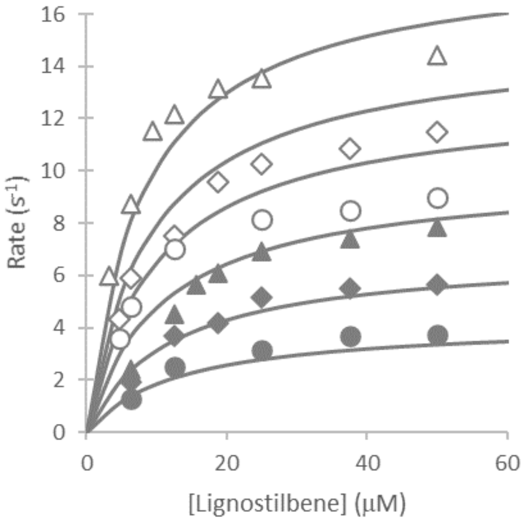

Figure S5. Michaelis-Menten plot of the inhibition of LSD4-catalyzed lignostilbene cleavage by vanillin. Experiments were performed using HEPES (I = 0.1 M), pH 7.5, at 25 °C, and 0 μM (△); 12.5 μM (◇); 25 μM (○); 50 μM (▲); 100 μM (◆); 200 μM (●) vanillin. The *lines* represent a best fit of an equation describing mixed inhibition to the data where K_{ic} = 30 ± 8 μM, K_{iu} = 60 ± 7 μM, K_M = 9 ± 1 μM, and k_{cat} = 18 ± 1 s^{-1}.

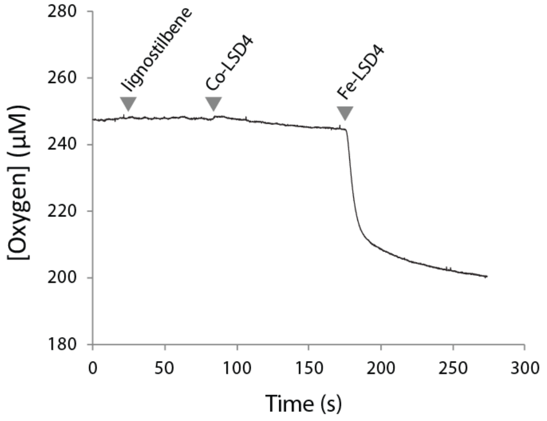

Figure S6. Activity comparison between Co and Fe-LSD4.
Progress curve in a standard oxygraph assay. Lignostilbene, Co-LSD4, and Fe-LSD4 were added at the indicated points. Enzymes were added to final concentrations of ~2 μM.

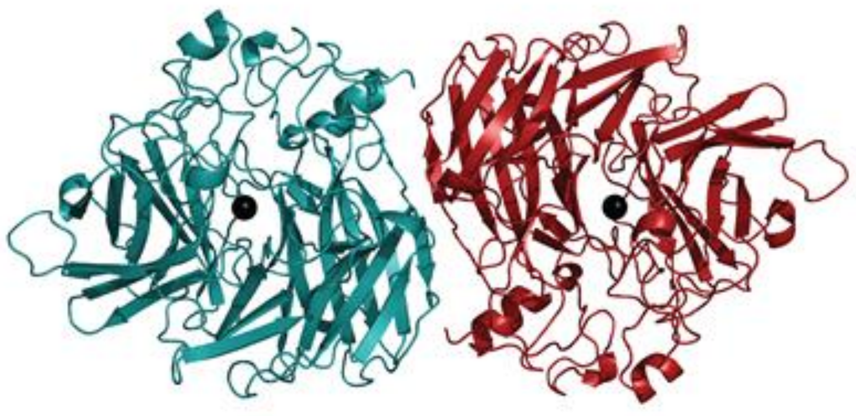

Figure S7. Structure of LSD4 dimer.
Ribbon and surface representation of the dimeric assembly of LSD4. The different protomers of the asymmetric unit and presumed dimer are teal and red, respectively. Fe^{2+} ions are shown as black spheres.

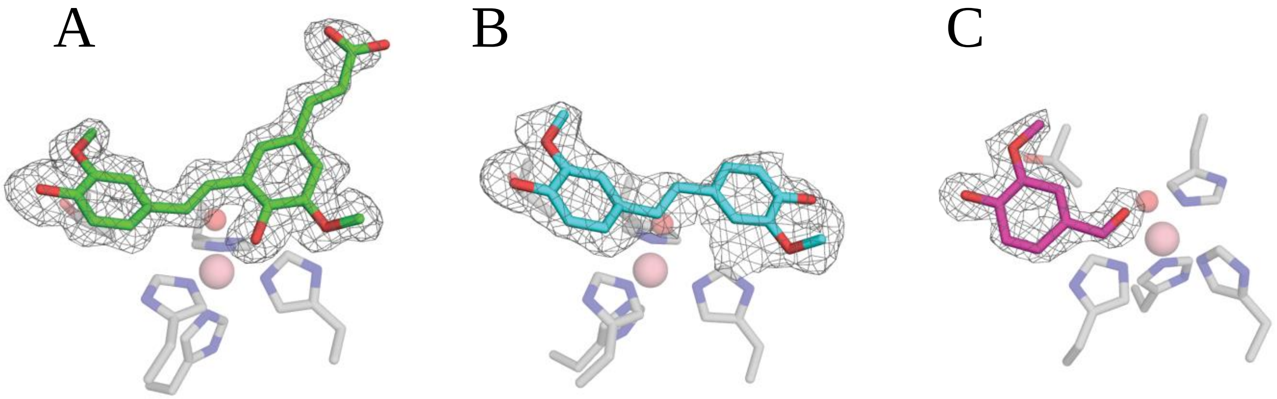

Figure S8. Omit density maps of Co-LSD4 complexes.
F_o-F_c electron density omit maps (contoured at 2σ and carved at 1.8 Å) of DCA-S (A), lignostilbene (B), and vanillin (C) for Co-LSD4 complexes. Active site metal and its coordinating residues are shown as a point of reference. LSD4 residues DCA-S, lignostilbene, and vanillin are presented as sticks and colored grey, magenta, cyan, and green, respectively. The cobalt ion and water molecule are modeled as pink and red sphere, respectively.

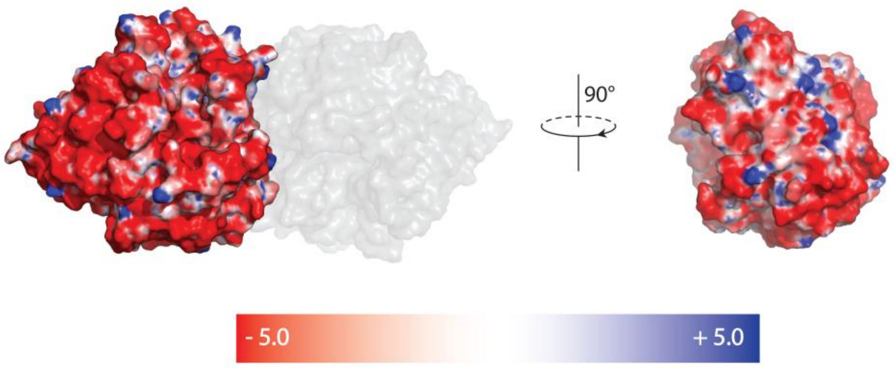

Figure S9. Surface electrostatic analyses of the inter-protomer interactions.
The analyses are shown as color-coded surface representations according to the degree of surface electrostatic charge which ranges from negative to positive (blue). Protomers of the dimeric partner is colored in gray at lowered opacity.

REFERENCES

1. Takahashi, K., Hirose, Y., Kamimura, N., Hishiyama, S., Hara, H., Araki, T., Kasai, D., Kajita, S., Katayama, Y., Fukuda, M., and Masai, E. (2015) Membrane-associated glucose-methanol-choline oxidoreductase family enzymes PhcC and PhcD are essential for enantioselective catabolism of dehydrodiconiferyl alcohol. *Appl Environ Microbiol* **81**, 8022-8036
